# Integrated physical, genetic and genome map of chickpea (*Cicer arietinum* L.)

**DOI:** 10.1007/s10142-014-0363-6

**Published:** 2014-03-08

**Authors:** Rajeev K. Varshney, Reyazul Rouf Mir, Sabhyata Bhatia, Mahendar Thudi, Yuqin Hu, Sarwar Azam, Yong Zhang, Deepa Jaganathan, Frank M. You, Jinliang Gao, Oscar Riera-Lizarazu, Ming-Cheng Luo

**Affiliations:** 1 International Crops Research Institute for the Semi-Arid Tropics (ICRISAT), Patancheru, India; 2 National Institute of Plant Genome Research (NIPGR), New Delhi, India; 3 University of California, Davis, USA; 4 Cereal Research Centre, Agriculture and Agri-Food Canada, Winnipeg, Canada; 5 Dow AgroSciences, Pullman, USA

**Keywords:** Chickpea, Physical map, Genetic maps, Reference genome sequence

## Abstract

Physical map of chickpea was developed for the reference chickpea genotype (ICC 4958) using bacterial artificial chromosome (BAC) libraries targeting 71,094 clones (~12× coverage). High information content fingerprinting (HICF) of these clones gave high-quality fingerprinting data for 67,483 clones, and 1,174 contigs comprising 46,112 clones and 3,256 singletons were defined. In brief, 574 Mb genome size was assembled in 1,174 contigs with an average of 0.49 Mb per contig and 3,256 singletons represent 407 Mb genome. The physical map was linked with two genetic maps with the help of 245 BAC-end sequence (BES)-derived simple sequence repeat (SSR) markers. This allowed locating some of the BACs in the vicinity of some important quantitative trait loci (QTLs) for drought tolerance and reistance to *Fusarium* wilt and *Ascochyta* blight. In addition, fingerprinted contig (FPC) assembly was also integrated with the draft genome sequence of chickpea. As a result, ~965 BACs including 163 minimum tilling path (MTP) clones could be mapped on eight pseudo-molecules of chickpea forming 491 hypothetical contigs representing 54,013,992 bp (~54 Mb) of the draft genome. Comprehensive analysis of markers in abiotic and biotic stress tolerance QTL regions led to identification of 654, 306 and 23 genes in drought tolerance “*QTL-hotspot*” region, *Ascochyta* blight resistance QTL region and *Fusarium* wilt resistance QTL region, respectively. Integrated physical, genetic and genome map should provide a foundation for cloning and isolation of QTLs/genes for molecular dissection of traits as well as markers for molecular breeding for chickpea improvement.

**Electronic supplementary material:**

The online version of this article (doi:10.1007/s10142-014-0363-6) contains supplementary material, which is available to authorized users.

## Introduction

Chickpea is a self-pollinated diploid (2*n* = 2*x* = 16) annual grain legume with a genome size of approximately 740 Mbp (Arumuganathan and Earle 1991). It is cultivated mostly on residual soil moisture in semi-arid regions of South Asia and sub-Saharan Africa. India is the largest producer of chickpea contributing about 60 % of total world’s production. Other important chickpea producing countries are Pakistan, Turkey, Mexico, USA, Canada and Australia (http://www.cgiar.org/our-research/crop-factsheets/chickpea/). The seeds of chickpea are rich in protein (24.6 %) and carbohydrate (64.6 %) and a good source of minerals and fibers and a source of food grain with low cholesterol levels (Abu-Salem and Abou 2011). There are two distinct chickpea types, *Desi* and *Kabuli*, different in their morphology. Desi-type chickpeas possess purple flower and small, dark and angular seeds and are largely consumed in Indian subcontinent and Pakistan, while *Kabuli* chickpeas have white flowers and large, cream-coloured seeds and are preferred in the Mediterranean basin and Central Asia where they are mainly consumed as a whole seed. Despite the increase in area and production during the last decade, there has not been a significant increase in the productivity of chickpea (Varshney et al. 2010). Considerable breeding efforts have been made across the globe to overcome the abiotic (drought, salinity, heat) and biotic (*Helicoverpa*, *Fusarium* wilt, *Ascochyta* blight) production constraints in chickpea. The genetic gains obtained through the conventional breeding efforts are not on par with the growing demands of the crop. Therefore, molecular breeding is now becoming one of the integral components of chickpea breeding programs (Chamarthi et al. 2011; Varshney et al. 2013a).

Recent advances in chickpea genomics have made it possible to not only develop large-scale molecular markers, genetic maps, transcriptomic resources for conducting large-scale and high-throughput marker genotyping but also sequence the genome of this important crop (Varshney et al. 2013b; Jain et al. 2013). For instance, >3,000 simple sequence repeat (SSR) markers (Nayak et al. 2010; Thudi et al. 2011; Choudhary et al. 2012), 15,360 Diversity Arrays Technology (DArT) loci (Thudi et al. 2011) and >3,000 single nucleotide polymorphism (SNP) markers (Hiremath et al. 2012; Gaur et al. 2012) have been developed. Several genetic maps have been constructed (Nayak et al. 2010; Millán et al. 2010; Gujaria et al. 2011; Thudi et al. 2011; Choudhary et al. 2012; Hiremath et al. 2012; Gaur et al. 2012), and hundreds of quantitative trait loci (QTLs) for drought tolerance (Varshney et al. 2014), salinity (Vadez et al. 2012), *Ascochyta* blight (Kottapalli et al. 2009; Anbessa et al. 2009), *Botrytis* grey mould (Anuradha et al. 2011) and *Fusarium* wilt (Tekeoglu et al. 2002; Udupa and Baum 2003) have been mapped. Further, large-scale expressed sequence tags (ESTs; Varshney et al. 2009) were generated and transcriptome assembly-constructed (Garg et al. 2011; Hiremath et al. 2012; Kudapa et al. 2014). In addition, several large-insert bacterial artificial chromosome (BAC) and binary bacterial artificial chromosome (BIBAC)-based libraries were also constructed for chickpea (Lichtenzveig et al. 2005; Zhang et al. 2010). The available genomic resources for chickpea molecular breeding community have been recently reviewed extensively by Upadhyaya et al. (2011) and Varshney et al. (2012).

In the context of development of physical maps, a BAC/BIBAC-based physical map was developed (Zhang et al. 2010). On this physical map, three large contigs closely linked to QTLs contributing to *Ascochyta* blight resistance and flowering time in chickpea were identified (Zhang et al. 2010). However, a comprehensive genome-wide physical map, and its integration with genetic maps possessing QTLs for important targeted traits and draft genome of chickpea, is the need of the hour for facilitating cloning of candidate genes and enhancing molecular breeding programs in this important crop.

In this study, we have constructed a genome-wide physical map employing large-insert BAC libraries and integrated it with the genetic maps and chickpea reference genome sequence for the identification of BAC clones covering important genomic regions for drought tolerance, *Ascochyta* blight and *Fusarium* wilt resistance. A comprehensive analysis of the targeted QTL regions in the integrated genetic, physical and genome maps has provided candidate genes that may be used for functional analysis as well as molecular breeding for drought tolerance and resistance to *Ascochyta* blight and *Fusarium* wilt.

## Materials and methods

### Plant material

ICC 4958, a drought tolerant *Desi* chickpea genotype, was used for the construction of BAC libraries. DNA was isolated from etiolated young seedling as per Cuc et al. (2008).

### BAC libraries

Two BAC libraries were constructed from high molecular weight (HMW) genomic DNA processed at Amplicon Express, Pullman, Washington, using the method of Tao et al. (2002). Purified large DNA fragments were ligated with pCCBAC1 (Epicentre, Omaha, NE, USA). Ligations were transformed into DH10B *Escherichia coli* cells (Invitrogen, Grand Island, NY, USA) and plated on Luria-Bertani (LB) agar with appropriate chloramphenicol, X-gal and IPTG concentrations. Clones were robotically picked with a Genomic Solutions G3 into 384-well plates containing LB freezing media. Plates were incubated for 18 h, replicated and then frozen at −80 °C. The replicated copy was used as a source plate for physical mapping.

### High information content fingerprinting

We used the four-colour high information content fingerprinting (HICF) SNaPshot method of Luo et al. (2003) with modification suited for ABI Genetic Analyzer 3730 platform (Gu et al. 2009). For each 384-well plate from the library, four 96-well blocks were inoculated using a 96-pin replicator. To control for plate orientation and fingerprinting, quality wells E7 and H12 were replaced in each 96-well block with a control BAC clone. The cultures were grown for 20 h at 37 °C and 400 rpm on an orbital shaker. The BAC DNA was purified using the Qiagen R.E.A.L. 96 Prep Kit (Qiagen, Valencia, CA, USA). Each BAC was simultaneously digested with four 6-bp recognizing restriction endonucleases generating 3′ recessed ends. Each of the four recessed 3′ ends was labeled with a different fluorescent dye using the SNaPshot kit (Applied Biosystems, Foster City, CA, USA). Restriction fragments were sized with a capillary DNA analyzer ABI 3730XL (Applied Biosystems, Foster City, CA, USA) using an internal GeneScan 1200 LIZ size standard. Fragment size calling was performed with the GeneMapper v. 3.7 (Applied Biosystems, Foster City, CA, USA).

### Fingerprinting data editing

Outputs of size-calling files from GeneMapper software were automatically edited with the FP Miner program (Gu et al. 2009). This software package was used to distinguish peaks corresponding to restriction fragments from peaks generated by a background noise in the profile of each BAC fingerprint and to remove vector restriction fragments from the profiles. The program also removed substandard profiles that could negatively affect contigs assembly. The files generated by FP Miner were used in the fingerprinted contig (FPC) assembly.

### Physical map contig assembly

Contigs were assembled from fragments within a size range of 70–1,000 bp using FPC software (version 9.3, http://www.agcol.arizona.edu/software/fpc/), using the following assembly strategy. We set tolerance at 6 (0.6 bp) and an initial cut-off of 1E − 60 (1 × 10^−60^). We followed the initial assembly with several rounds of DQer, until no contig containing 15 % or more questionable (Q) clones. This was followed by several rounds of end-to-end merging and single-to-end merging at progressively lower cut-off stringencies. The “best of” function was set to 100 builds.

FPC displays the length of each BAC clone and each BAC contig in consensus band (CB) units. To convert CB units to kilobytes, we estimated the lengths of inserts in 100 BAC clones in kb by pulsed field gel electrophoresis (PFGE) and divided the total length by the number of restriction fragments in the fingerprints of the clones. The conversion factor for the BAC clones was 1.92 kb/CB unit.

### Anchoring of physical map with genetic maps

For anchoring the physical map with the genetic map, a set of 337 BAC clones corresponding to 337 polymorphic BAC-end sequence-derived SSR (BES-SSR) markers in inter-specific genetic map (ICC 4958 × PI 489777; Thudi et al. 2011) and two intra-specific genetic maps (ICC 4958 × ICC 1882 and ICC 283 × ICC 8261) were subjected to fingerprinting in the same way as mentioned above. The fingerprinted clones were tried for their assembly with the FPC contig physical map for anchoring genetic map with the physical map.

### Alignment of physical map with draft genome of chickpea

A set of 4,290 BAC clones forming the minimum tilling path (MTP) of FPC contig were picked, and plasmid DNA was isolated as described by Huo et al. (2008). BAC clones were sequenced with BigDye Terminator v3.1 (Applied Biosystems, Foster City, CA, USA) using the pIndigoBAC-5 reverse primer (5′-CTCGTATGTTGTGTGGAATTGTGAGC-3′). Amplifications were carried out in 96-well plate (10 μl reaction volumes containing 1 μl of BigDye, 1.75 μl sequencing reaction buffer, 2 mM MgCl_2_, 5 % DMSO, 0.5 μM primer and 200–500 ng of BAC DNA) or 384-well plate (5 μl reaction volume). PCR was performed with 49 cycles of 96 °C for 10 s, 50 °C for 10 s and 60 °C for 4 min. The PCR product was purified and then analyzed on the 3730XL DNA analyzer (Applied Biosystems, Foster City, CA, USA). Base calling of ABI trace file was performed with PHRED (Ewing and Green 1998; Ewing et al. 1998), and sequences with quality scores of less than 20 were trimmed. Vector sequences were removed using Cross_match. BES less than 100 bp was also filtered.

Similarly, another set of 46,270 BAC-end sequences for CAH1 library that were used to develop novel SSR markers and development of high-density genetic map in chickpea were also used for in silico mapping onto draft chickpea genome during the present study. The details of construction of BAC library, BAC-end sequences is available elsewhere (Thudi et al. 2011).

In addition, efforts have also been made to map onto draft genome sequence the genetically mapped 1,328 marker loci including novel 625 Chickpea KASPar Assay Markers (CKAMs) and 314 tentative orthologous genes (TOGs)-SNPs from Hiremath et al. (2012) for integrating physical, sequence and genetic maps. For repeat analysis, a repeat database for chickpea genome was first built from the *Kabuli* draft genome already available with us (Varshney et al. 2013b), followed by search for different types of repeat sequences from the 53,316 BESs using RepeatMasker v 3.2.7 (Tarailo-Graovac and Chen 2009). The unique sequences were isolated from repetitive ones using NCBI BLAST+ ver 2.2.25 with a cut-off *E* value of less than 10^−50^. All the 53,316 BESs were used as Basic Local Alignment Search Tool (BLAST) query against chickpea repeat database.

The clean sequences after repetitive sequence analysis were subjected to mapping onto draft genome of *Kabuli* chickpea using NCBI BLAST+. From all of the BLAST alignments, clean BESs were extracted according to the following criteria (Katagiri et al. 2004; Asamizu et al. 2012): (i) sequence identity >90 % and alignment coverage >50 %, (ii) mapped positions of each pair of ends >100 bp and <200 kb apart in the same chromosome, (iii) direction of each paired end is correct, (iv) BLASTN *E* < 10^−100^, (v) a minimum of one hit for one of the paired ends and (vi) no redundant chromosomal locations.

### Identification of genes in important QTL regions

In order to identify candidate genes present in the QTL regions for some important traits such as drought tolerance (“*QTL-hotspot*”, Varshney et al. 2014), *Ascochyta* blight resistance (Udupa and Baum 2003) and *Fusarium* wilt resistance (Sabbavarapu et al. 2013), the markers present in these QTL regions were subjected to BLAST against chickpea genome assembly (Varshney et al. 2013b) and the corresponding UniProt IDs were retrieved. For functional categorization of the genes, the UniProt IDs of the genes were mapped onto UniProt KB database (http://www.uniprot.org/).

## Results

### New BAC libraries

**Fig. 1:**
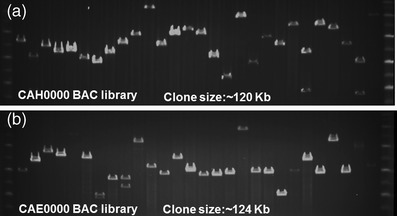
Insert size estimation of chickpea BAC clones by pulsed field gel electrophoresis (PFGE). A set of 28 clones can be visualized from **a** CAH0000 library (constructed using *Hin*dIII) and **b** CAE0000 library (constructed using *Eco*RI). PFG marker can be seen in two lanes on either side of the 28 clones from each library. The insert size of BAC library is ~120 and ~124 kb for CAH0000 and CAE0000 libraries, respectively

Two BAC libraries were constructed from genomic DNA of chickpea *Desi* cultivar “ICC 4958” using *Hin*dIII and *Eco*RI restriction enzymes and were designated as CAH0000 and CAE0000, respectively. From each of these two libraries, 48,384 clones were picked up, and in total, 96,768 clones were obtained. For quality control, four plates were randomly selected from each library, and 24 clones from various quadrants of each plate were picked up. In brief, 96 random clones were selected from both CAE0000 and CAH0000 BAC libraries for quality control analysis. These analyses revealed average insert sizes of 120 kb in CAH0000 library and 124 kb in CAE0000 library and approximately eight-fold coverage of the chickpea haploid genome in each of these two libraries (Fig. 1a, b). It was also observed that ~3 to 4 % of clones in both libraries contained no inserts of chickpea DNA.

In addition, another BAC library (CAH1) constructed from the chickpea cultivar ICC 4958 using *Hin*dIII restriction enzyme (Thudi et al. 2011) was also used in the present study. From this library, a set of 1,110 BACs containing resistance gene homolog (RGH) and BES-SSRs were used for developing the physical map.

### BAC fingerprinting

A set of 71,094 clones (~12× coverage) including 35,040 clones from CAH0000, 34,944 clones from CAE0000 library, 337 BES-SSR-containing clones and 773 disease resistance gene-containing clones from CAH1 library (Thudi et al. 2011) were targeted for fingerprinting (Table 1). Fingerprints were obtained for 68,248 clones (95.99 %). After removing cross-contaminated clones, clones with imperfect fingerprints and clones possessing small inserts, 49,368 clone fingerprints were found to be suitable and were used for contig assembly (Table 1). Number of bands from each clone varied from 34 to 2,268 (Table 2).

**Table 1:** Statistics of BAC fingerprinting of different libraries in chickpea

Clone statistics	CAH library	CAE library	CAH1 library	Total
Clones targeted	35,040	34,944	1,110	71,094
Clones with usable fingerprinting data	33,652	33,512	1,084	68,248
Clones in FPC	21,787	26,497	1,084	49,368

**Table 2:** Summary of statistics of the chickpea physical map

Feature	Statistic
Total no. of BAC clones targeted	71,094 (~12× coverage)
No. of BAC clones with usable data	68,248
Contig assembly results	
No. of clones in assembly	49,368
No. of clones in contigs	46,112
No. of clones as singletons	3,256 (~300 Mb)
No. of contigs	1,174
Longest contig	4.1 Mb
Total contiguous coverage	615 Mb
Contig size distribution (no. of contigs by group)	
No. of contigs with 2 clones	88
No. of contigs with 3–9 clones	366
No. of contigs with 10–24 clones	311
No. of contigs with 25–49	188
No. of contigs with 50–99 clones	120
No. of contigs with 100–199 clones	69
No. of contigs with ≥200 clones	32
Total	1,174
Q clones statistics in contigs	
No. of contigs without Q clones	731
No. of contigs with Q clones	443
Total no. of Q clones	2,093
Range of Q clones in contigs	1 to 89
Clone statistics in contigs	
Total no. clones in 1,174 contigs	46,112
Range of clone in contigs	2 to 3,007
Average no. of clones in each contig	39.27
Genome coverage (total clones ×130 kb)	8×
Band statistics in clones	
Total no. of bands in clones	318,971
Average no. of bands in clones	271.69
Range of bands in clones	34 to 2,268
Minimum tilling path (MTP)	
Total no. of clones in contigs	4,290
Genome represented	503 Mb

### Physical map assembly

**Fig. 2:**
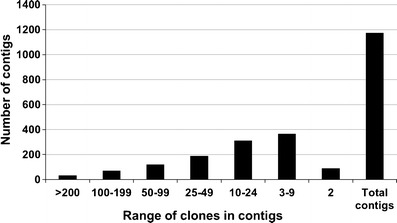
Distribution of number of clones in 1,174 contigs in chickpea FPC assembly. The range of clones in each contig varied from 2 to 3,007 with an average of 39.27 clones/contig

FPC version 9.3 was used for assembling a physical map. A total of 1,174 contigs could be assembled from 49,368 clones with usable fingerprinting data with the procedure described in the “Materials and methods” section (Table 2). The 1,174 contigs contained 46,112 clones, of all of which, a set of 4,290 clones defined the MTP of physical map assembly, while 3,256 clones remained as singletons. The range of clones in each contig varied from 2 to 3,007 with an average of 39.27 clones/contig (Table 2, Fig. 2; http://phymap.ucdavis.edu/chickpea/).

An analysis of Q clones in our data set indicated that 443 contigs out of total 1,174 contigs (37.73 %) contained a total of 2,093 Q clones (2,093 / 49,368 = 4.23 %) with a range of 1 to 89 Q clones/contig (Table 2). The low number (4.23 %) of Q clones in our assembly indicated the good quality and proper cut-off and tolerance values used for the assembly.

### Anchoring physical map with genetic maps

In order to anchor the contig map with the genetic map, a total of 337 polymorphic BES-SSRs were used for mapping on three mapping populations (one inter- and two intra-specific crosses). However, only 259 BES-SSR markers were integrated on genetic maps based on three populations, namely, ICC 4958 × PI 489777 (inter-specific mapping population) and ICC 4958 × ICC 1882 and ICC 283 × ICC 8261 (intra-specific populations) (Supplementary Table 1). A consensus map was also developed based on genetic maps for intra-specific mapping populations (http://cmap.icrisat.ac.in/cmap/sm/cp/varshney/).

**Fig. 3:**
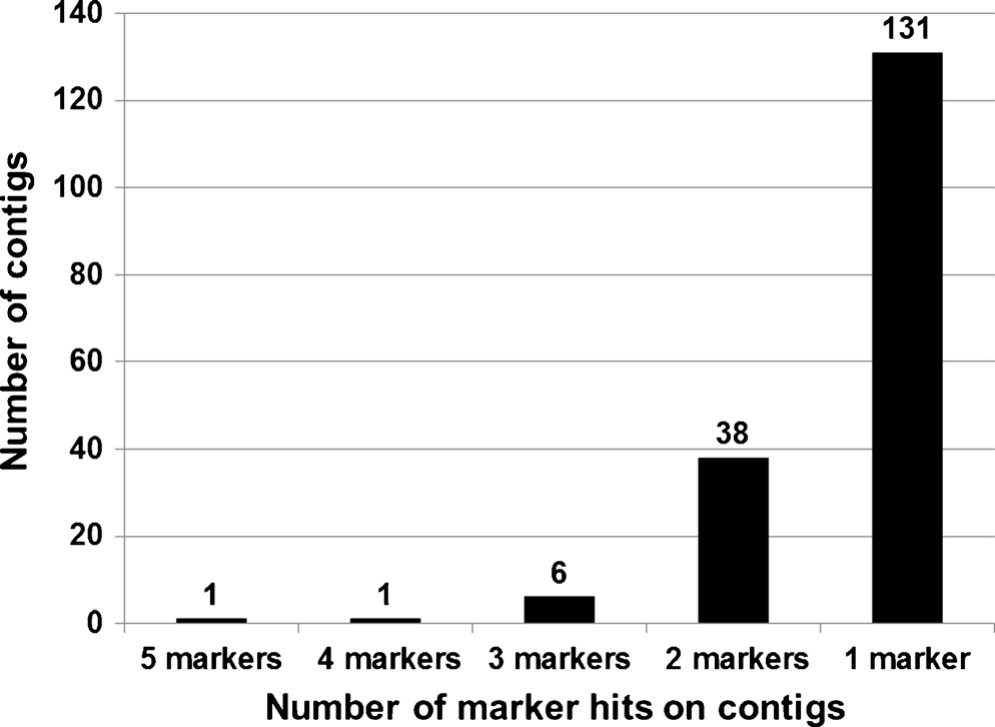
Anchoring physical map with two chickpea genetic maps. The number of marker hits on BAC contigs varied from 1 to 5; unique hits were obtained for 131 markers

BAC clones of all 337 polymorphic BES-SSRs were subjected for fingerprinting as mentioned above, and 319 were assembled into FPC assembly (Table 1). However, only 259 mapped markers on above-mentioned genetic maps could be aligned onto the physical map. These data were used for anchoring genetic maps with the physical map (Supplementary Table 1). A summary of distribution of these 259 mapped markers on different linkage groups (LGs) is tabulated in Table 3. The details of contigs hit by these BES-SSRs of chickpea FPC assembly have been given in Supplementary Table 1 and in Fig. 3.

**Table 3:** Distribution of 259 BES-SSR markers mapped on FPC assembly of chickpea

Linkage group	Number of markers mapped		Total
	Inter-specific map (ICC 4958 × PI 489777; Thudi et al. 2011)	Consensus map (ICC 4958 × ICC 1882 and ICC 283 × ICC 8261; Varshney et al. 2014)	
CaLG01	6	8	14
CaLG02	3	5	8
CaLG03	34	32	64
CaLG04	17	13	29
CaLG05	32	15	45
CaLG06	25	19	40
CaLG07	33	12	42
CaLG08	7	10	17
Total	157	114	259

In addition to above, 619 genetically mapped marker loci including CKAMs and TOGs that were mapped onto chickpea draft genome sequence/scaffolds were found very close/linked to all 163 MTP clones of physical map mapped onto eight pseudo-molecules of draft genome sequence. This has also helped to anchor a physical map with the second-generation genetic map developed based on cost-effective SNP marker assays (Hiremath et al. 2012).

### QTLs assigned to integrated genetic and physical map

A number of QTLs were identified for abiotic and biotic stresses earlier; in addition, we also identified QTLs for abiotic stress (drought tolerance, salinity) and biotic stress (like *Fusarium* wilt, *Ascochyta* blight and *Botrytis* grey mould) from published studies, and their corresponding location on the inter-specific map based on ICC 4958 × PI 489777 (Thudi et al. 2011) was determined (Supplementary Table 1).

**Fig. 4:**
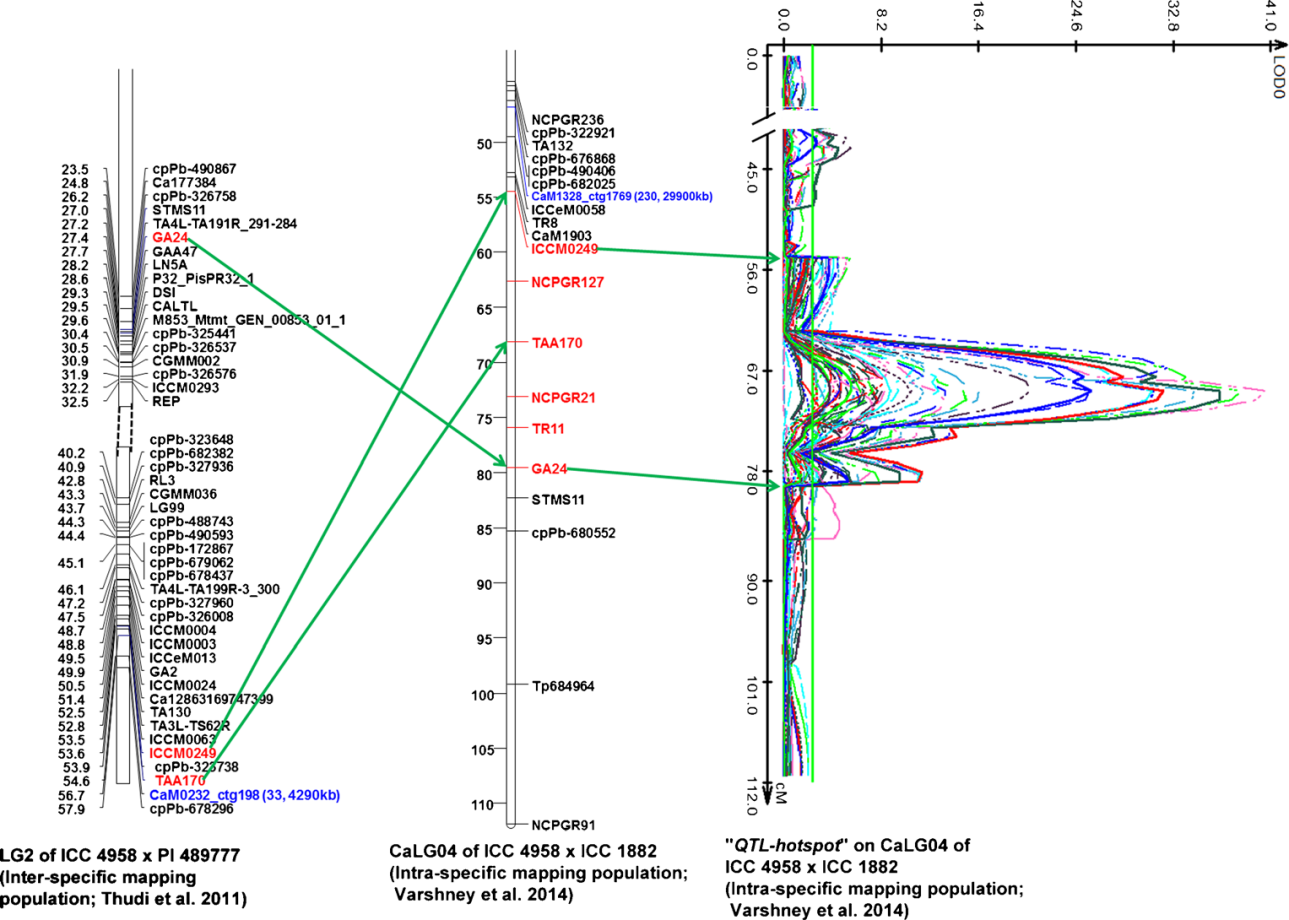
A snapshot of anchoring BAC clones to the “*QTL-hotspot*” region harbouring QTLs for several drought tolerance-related traits in chickpea. Two BAC contigs namely ctg198 and ctg1769 were assigned to “*QTL-hotspot*” region as SSR markers namely CaM0232 and CaM1328 derived from end sequences of BACs from the above mentioned contigs were mapped in the same region on inter-specific (Thudi et al. 2011) and intra-specific (Varshney et al. 2014) genetic maps, respectively

While analyzing the physical map in details, several BAC clones in proximity to "*QTL-hotspot*" were identified on CaLG04, and its traits were identified. The “*QTL-hotspot*” possesses QTLs for root traits (root length density, root volume, root surface area, root length, root dry weight, shoot dry weight, leaf dry weight), phenological traits (days to flowering and days to maturity), yield, harvest index, biomass, 100-seed weight, seeds per pod and carbon isotope discrimination under both rain-fed and irrigated conditions (Varshney et al. 2014). A BAC contig (ctg198) has been found very close (2.1 cM) to the associated/linked marker TAA170 on the intra-specific genetic map of ICC 4958 × ICC 1882, and BAC contig (ctg1769) was found very close to ICCM0249 markers on the linkage group CaLG04 of an inter-specific genetic map of ICC 4958 × PI 489777 (Fig. 4).

**Fig. 5:**
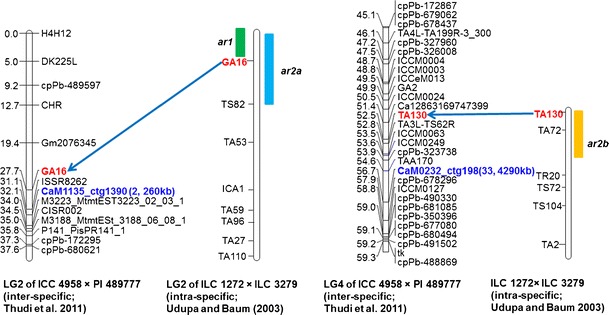
A snapshot showing coverage of *Ascochyta* blight resistance QTL regions with BAC clones in chickpea. The BAC ctg1390 on LG2 of inter-specific genetic map developed by Thudi et al. (2011) based on RIL population ICC 4958 × PI 489777 is closer to the SSR marker *GA16* that flanks the *ar2a* QTL identified for *Ascochyta* blight resistance by Udupa and Baum (2003). Similarly, the BAC contig 198 on LG04 of inter-specific genetic map developed by Thudi et al. (2011) is closer to SSR marker TA130 that flanks *ar2b* QTL identified for *Ascochyta* blight resistance by Udupa and Baum (2003)

A BAC contig closer to the *Ascochyta* blight resistance QTLs reported by Aryamanesh et al. (2010) and Kottapalli et al. (2009) was identified. Similarly, several other BAC contigs were found to be closer or in the same QTL regions. For instance, ctg1390 (260 kb) was found closer to *ar1* and *ar2a* on LG02, QTLs for *Ascochyta* blight resistance, and similarly, ctg298 (4,290 kb) was found close to *ar2b* QTL on LG04 reported by Udupa and Baum (2003) (Fig. 5). One contig, ctg248, was identified in close proximity to the *Fusarium oxysporum* f. sp. *ciceris race 0* locus reported by Cobos et al. (2007). Fourteen contigs were also found in close proximity to the beta-carotene-related QTLs (Supplementary Table 1).

### Anchoring physical map with the draft genome of *Kabuli* chickpea

**Fig. 6:**
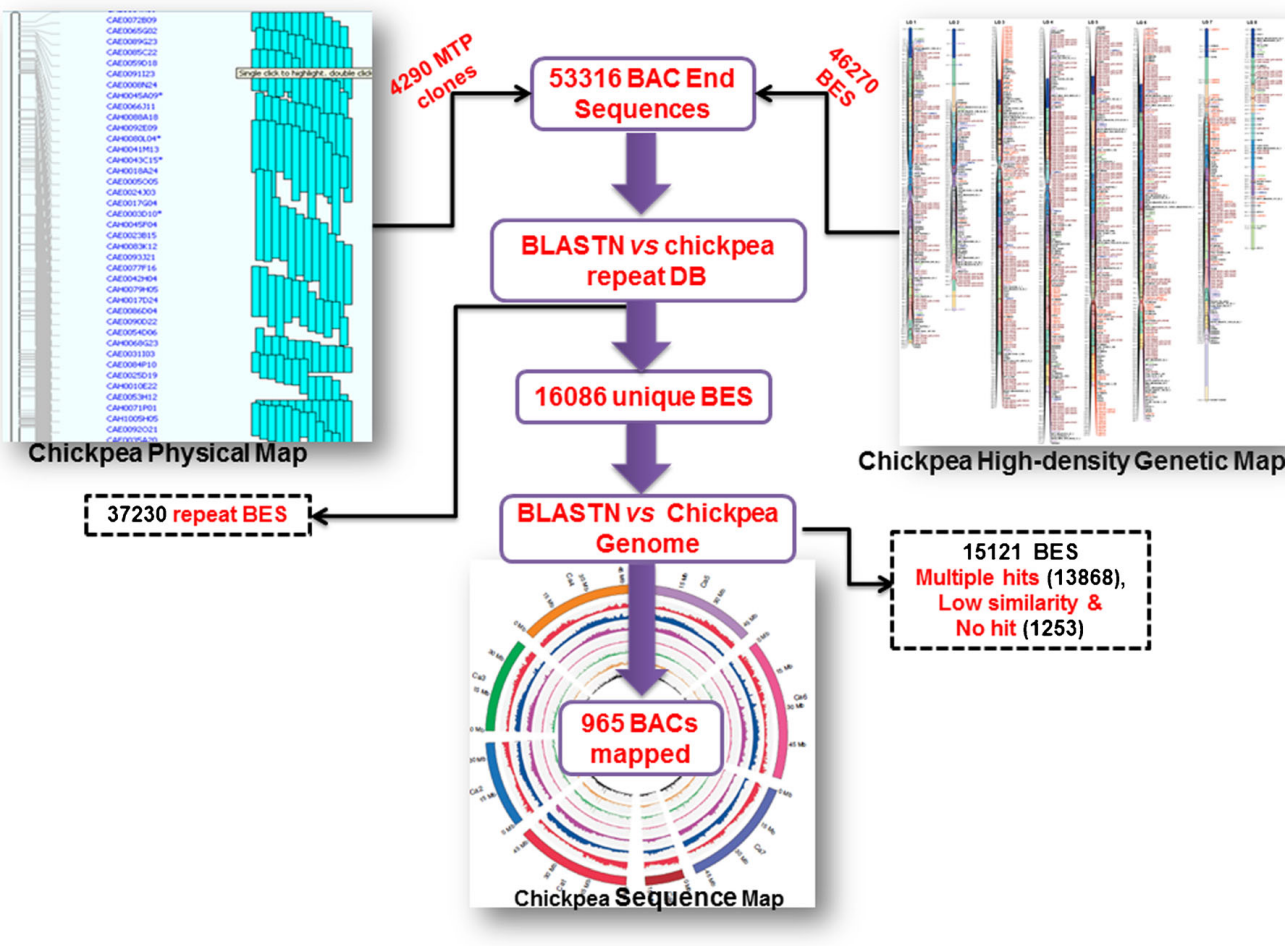
Summary of steps involved in BES mapping analysis for integrating chickpea FPC contig assembly with the high-density genetic map and the reference chickpea genome sequence. A set of 53,316 BESs from 28,147 clones were used for anchoring contig physical map with the draft genome sequence of chickpea. BLASTN analysis of 53,316 BESs provided 16,086 unique (non-repeat) BES. The unique BESs (16,086) were tried for their mapping onto the chickpea genome sequence covering eight pseudo-molecules (Ca1 to Ca8) and un-anchored scaffolds (Ca0). As a result, a total of 965 BAC clones were successfully mapped onto draft genome of *Kabuli* chickpea, while 13,868 BES had multiple hits, and 1,252 had either low similarity or no hits

For anchoring a contig physical map with the draft genome sequence of chickpea (sequence map; Varshney et al. 2013b), a set of 53,316 BESs (37.8 Mb including redundancy) from 28,147 clones representing ~5.12 % chickpea genome were used (Table 4). BESs ranged from 64 to 1,113 bp with an average of 708 bp. Among 53,316 BESs, 50,338 BES (94 %) were found to be mate pairs from 25,169 clones and 2,978 BESs (6 %) were single ends of BAC clones (Table 4). The repeat analyses of all the BESs showed ~70 % of sequences which constitute different kinds of repeats with abundance (~60 %) of LTR elements (Supplementary Table 2). Further analysis of BESs provided 16,086 unique (non-repeat) BES, while 37,230 BESs were found to contain repeat BESs and were therefore excluded from further mapping (Fig. 6). The unique BESs (16,086; 30 %) were tried for their mapping onto the chickpea genome sequence covering eight pseudo-molecules (Ca1 to Ca8) and un-anchored scaffolds (Ca0). A total of 965 BAC clones could be successfully mapped onto a draft genome of *Kabuli* chickpea (Table 5; Supplementary Fig. 1a–h). The mapped BAC clone size distribution on the basis of results revealed 428 out of 965 (44.35 %) BACs ranged from 90 to 110 kb with an average size of 95 kb. The number of BAC clones mapped on individual pseudo-molecule ranged from 64 on Ca8 to 154 on Ca4 with an average of ~110 BAC clones/pseudo-molecule (Table 5). A set of 85 BACs were mapped on scaffolds not assigned to any pseudo-molecule (Ca0). The number of BAC clones mapped on chickpea genome sequence consists of 491 contigs covering a total of ~55 Mb physical length (Table 5). The number of contigs on each pseudo-molecule ranged from 31 on Ca8 to 93 on Ca4 with an average of ~62 contigs/pseudo-molecule (Table 5). All these contigs represent only a small proportion (~15 %) of the genome represented by eight pseudo-molecules.

**Table 4:** Results of BAC-end sequencing of ICC 4958

Feature	Number
Total BAC clones of MTP	4,290
No. of BES generated	7,046
No of BAC clones targeted from CAH1 library	25,000
No. of BES generated	46,270
Total number of clones for in silico mapping	28,147
No. of BES generated	53,316
Genome represented	37.8 Mb (including redundant clones)
Mate pairs seq	50,338 (25,169)
Single-end seq	2,978 (2,978 clones)

**Table 5:** Results of in silico mapping of BAC clones on the chickpea reference genome

Pseudo-molecule	Length of pseudo-molecule (Mb)	Mapped BACs^a^	No. of contigs	Pseudo-molecule coverage (bp)
Ca1	48.36	134 (23)	61	7,098,345
Ca2	36.63	65 (20)	48	5,030,030
Ca3	39.99	114 (16)	53	615,545
Ca4	49.19	154 (29)	93	10,319,178
Ca5	48.17	106 (21)	65	6,950,946
Ca6	59.46	129 (26)	75	8,317,695
Ca7	48.96	113 (17)	65	6,798,926
Ca8	16.48	64 (11)	31	3,323,327
Total	347.24	879 (163)	491	54,013,992

^a^Number in parenthesis indicate the number of MTP clones

Similarly, we also tried to map sequences of 1,328 genetically mapped loci from Hiremath et al. (2012) onto the daft genome sequence of chickpea. As a result, 619 CKAMs and TOGs were mapped onto the chickpea draft genome sequence/scaffolds and were found to be very close/linked to MTP clones of a physical map mapped onto draft genome sequence. The number of mapped marker loci on the draft genome sequence ranged from 43 (on Ca8) to 113 (on Ca4) with an average of ~77 loci/pseudo-molecule (Supplementary Fig. 1a–h). This helped us to anchor a physical map with the draft genome sequence of chickpea and genetic maps.

### Mining candidate genes in select stress responsive QTL regions

After identification of BAC contigs/clones in the region or proximity QTLs for drought tolerance (“*QTL-hotspot*”), *Ascochyta* blight and *Fusarium* wilt resistance, an effort was made to mine candidate genes in the genomic regions after aligning molecular markers present in/associated with the QTL regions on genome map/sequence assembly. For instance, in the case of “*QTL-hotspot*” region, analysis of seven SSR markers (ICCM0249, NCPGR127, TAA170, NCPGR21, TR11, STMS 11 and GA24) on CaLG04 with a genome sequence identified a region of ca. 7 Mb (starting at 9.1 Mb and ending at 16.1 Mb) on Ca4 chromosome. Genome annotation of this region has identified a total of 654 genes (Fig. 5; Supplementary Table 3). In the case of *Ascochyta* blight resistance, analysis of two SSR markers (GA16 and CaM1135) present in QTL region/genes *ara*1 and *ara*2a (Thudi et al. 2011) identified a 3 Mb region (starting at 31 Mb and ending at 34 Mb) on Ca2 that contained a total of 306 genes (Supplementary Table 4). However, the markers TA130 and CaM0232 on LG4 (Thudi et al. 2011; Fig. 5) corresponding to *ara*2b QTL/gene could not be mapped; hence, this QTL region was not analyzed in details. For *Fusarium* wilt resistance QTL region, analysis of two markers (CaM1125 and TR44; Sabbavarapu et al. 2013) on genome sequence provided a relatively smaller region (0.6 Mb, between 29 and 29.6 Mb), comprising 23 genes (Supplementary Table 5).

All 983 genes (654 in “*QTL-hotspot*” region, 306 in *Ascochyta* blight resistance QTL region and 23 in *Fusarium* wilt resistance QTL region) were functionally categorized based on Gene Ontology (GO) descriptions (UniProt database, 151 UniProt-GO), and all could be assigned to at least one GO term (Table 6). The genes in all three QTL regions were further assigned to three functional categories: (i) molecular function, (ii) cellular component and (iii) biological processes.

**Table 6:** Functional categorization of genes present in the QTL regions of drought tolerance ("*QTL-hotspot*"), *Fusarium* wilt and *Ascochyta* blight resistance

Functional category	Number of genes in different QTL regions		
	“*QTL-hotspot*” (Varshney et al. 2014)	*Ascochyta* blight resistance *ara1* and *ara2a* (Udupa and Baum 2003)	*Fusarium* wilt resistance (Sabbavarapu et al. 2013)
Molecular function	362	157	14
Catalytic activity	239	95	6
Binding	219	92	11
Transcription factor binding transcription factor activity	3	-	2
Structural molecule activity	14	5	-
Transporter activity	24	13	-
Electron carrier activity	1	5	-
Enzyme regulator activity	3	3	-
Sequence-specific DNA binding transcription factor activity	21	8	-
Peroxidase activity	2	1	-
Signal transducer activity	2	-	-
Nutrient reservoir activity	2	-	-
Superoxide dismutase activity	-	-	1
Structural constituent of ribosome	-	-	1
MAP kinase activity	-	1	-
Cellular component	252	112	9
Cell part	190	88	9
Organelle part	43	15	2
Organelle	160	64	7
Membrane	93	42	3
Membrane part	53	26	-
Extracellular region	16	4	-
Plasmodesma	3	1	-
Apoplast	-	-	1
Extracellular space	1	-	-
Macromolecular complex	31	18	-
Nuclear lumen	5	2	-
Virion part	-	2	-
Ribosome	-	-	1
Biological process	369	162	14
Cellular process	239	103	12
Response to stimulus	54	27	4
Localization	47	28	3
Establishment of localization	47	27	3
Metabolic process	296	118	10
Single-organism process	103	56	7
Cellular component organization or biogenesis	30	18	2
Reproduction	19	10	3
Multicellular organismal process	31	-	2
Cell adhesion	2	2	-
Reproductive process	18	9	-
Developmental process	26	14	-
Growth	2	2	-
Multiorganism process	8	3	-
Biological regulation	77	35	-
Immune system process	2	-	-
Single organism signaling	10	-	-
Developmental process involved in reproduction	-	-	2
Defense response to bacterium, incompatible interaction	-	-	1
Single-multicellular organism process	-	-	2
Regulation of biological process	-	-	3
Response to other organism	-	-	1
Systemic acquired resistance	-	1	-
Signal transduction	-	11	-
Multicellular organismal process	-	14	-

The sum of a number of genes in different classes in a given functional category is higher than the total number of genes assigned to a particular functional category as a given gene may be associated with different classes of the respective functional category

In the case of “*QTL-hotspot*” region, out of 654 genes, 362 genes were assigned to “molecular function” category, 252 genes to “cellular component” category and 370 genes to “biological process” category, respectively. It is important to note here that the sum of genes assigned to different functional categories (984) is higher than the total number of genes (654), as a given gene may fall in more than one category. In the molecular function category, the highest number of genes fell into catalytic activity (239) followed by binding (219). Under cellular component category, the highest number of genes fell into cell part (190) followed by organelle (160). Similarly, in the biological processes category, a maximum number of genes fell into metabolic process (296) followed by cellular process (239).

For *Ascochyta* blight QTL region, 157 genes were assigned to molecular function, 112 genes to cellular component and 162 genes to biological processes. The highest number of genes under molecular function category fell into catalytic activity (95). In the cellular component category, a maximum number of genes (88) belonged to cell part class. Similarly, under biological processes category, the highest number of genes fell to metabolic process class that contained the highest number of genes (118).

In the case of *Fusarium* wilt resistance QTL region, 14 genes were assigned to molecular function category, 9 genes to cellular component category and 14 genes to biological processes, respectively. Among these categories, binding class with 11 genes, cell part with 9 genes and cellular process with 12 genes were found in abundance under molecular function, cellular component and biological process categories, respectively.

## Discussion

Chickpea is one of the important diploid legume crops (with moderate genome size of 738 Mb) for the poor, largely vegetarian people living in semi-arid tropics (SAT) and other climate vulnerable regions in the world. The availability of physical maps in crop plants serves as an indispensable tool and have been used for a variety of purposes including (i) QTL fine mapping and positional (map-based) cloning of QTLs/genes (Bakker et al. 2003), (ii) anchoring chromosomes using fluorescence in situ hybridization (FISH) (Islam-Faridi et al. 2002), (iii) repeat classification (Cardle et al. 2000), (iv) draft genome sequence assembly (Sasaki et al. 2005), (v) marker development experiments (van der Vossen et al. 2000) and (vi) analysis of structural variation in the genome (Kidd et al. 2008). In the recent past, significant progress and tremendous advancements have been made in the area of sequencing/resequencing crop genomes using next-generation sequencing (NGS) technologies (Hillier et al. 2008; Wheeler et al. 2008; Thudi et al. 2012). However, despite the advancements in NGS technologies, the need and value for development of high-quality physical maps still remains high (Lewin et al. 2009). For instance, the sequencing/assembly of crops possessing complex genomes with large fractions of repetitive genome will not be easily addressed by NGS alone but will be facilitated by physical maps which would provide anchor points to link sequence contigs and bridge gaps due to large repeat regions. One of the effective ways for providing these anchor points is by construction of a whole genome physical map using bacterial artificial chromosome (BAC) clones (Shizuya et al. 1992; Rounsley et al. 2009) and BAC-end sequencing (Nelson et al. 2005). The development of physical maps using BAC clones has been proven effective for the construction of genome-wide physical maps, since generation and storage of these clones is somewhat relatively easy (Gregory et al. 1997; Marra et al. 1997; Klein et al. 2000; Wu et al. 2004). Therefore, BAC-based physical maps combined with BAC-end sequencing have formed the basis of several whole genome sequencing projects (Sasaki et al. 2005; Wei et al. 2007). Keeping the importance of physical maps in view, a BAC-based physical map has been developed for chickpea in the present study.

### Physical map assembly and genome coverage/representation of chickpea genome

In the present study, we reported the construction of a high-quality, high-coverage BAC-based physical map of chickpea genome using genomic DNA of cultivar ICC 4958. The physical map has a total of 49,368 BAC clones assembled into 1,174 contigs. The number of clones in each contig varied from 2 to 3,007 with an average of ~39 clones/contig spanning from >200 kb to 4.1 Mb, with an average physical length of ~489 kb (574 / 117 = 0.4889 Mb). These contigs collectively span 615 Mb, smaller than the expected 738 Mb estimated genome size of chickpea genome by 17 %.

The genome coverage (eight times) of chickpea genome (46,112 clones) assembled into chickpea physical map assembly (Table 2) was sufficient. Comparative genome coverage (10× coverage) has been found earlier to be sufficient for developing a physical map of the chickpea genome (Zhang et al. 2010). Similarly, the number of clones used for fingerprinting (70,321) has been also found sufficient for generating a quality physical map, since less number of clones (67,584) were fingerprinted in a previous study of chickpea physical map development (Zhang et al. 2010).

The chickpea physical map was developed using two BAC libraries based on patterns of fragments generated by digestion of restriction enzymes (*Bam*HI, *Eco*RI, *Xba*I, *Xho*I and *Hae*III). The genome size estimated by K-mer analysis revealed a genome size of 738 Mb. However, genome size of only 615 Mb could be assembled into contigs, while 300 Mb remained in the form of singletons. The estimation of coverage of physical map was based on contigs only, and if singletons were also included, then the genome coverage of chickpea physical map might be overestimated (615 + 300 = 915 Mb). The discrepancy in genome size estimation is not unusual as in earlier studies; also, overestimation of genome size has been reported such as in *Populus* (about 20 % larger; Kelleher et al. 2007) and soybean (26.3 % larger; Wu et al. 2004). However, in the case of papaya, the genome size estimated based on the physical map was close to the actual size (Yu et al. 2009). The smaller genome size obtained in the present study may be attributed to the following: (i) false end-merges of clones and formation of large contigs; (ii) more overlaps between contigs; (iii) 3,256 or 6.5 % of clones remained as singletons, and 4.4 % (2,137) of the clones were located on small contigs (containing fewer than ten clones); (iv) average insert size estimation was not perfect based on representative samples of BAC libraries; (v) genome size of chickpea not perfectly estimated earlier; and (vi) the use of only two restriction enzymes for BAC library construction, since the use of more number of RE in BAC library construction has been found to increase the actual coverage of map (Zhang et al. 2010). The existence of more overlap than the expected between the contigs will reduce the overall size of contigs and, hence, the physical map assembly. Discrepancies in genome size of chickpea was also reported in an earlier study of the development of physical map in chickpea; however, they reported more genome size than the expected 738 Mb (Zhang et al. 2010). Despite the genome size estimation discrepancy, the quality of the chickpea physical map developed in the present study was found to be sufficiently high for use in various applications of genomics research.

### Integrating physical map with the genetic/QTL maps

A genetic linkage map is constructed by placing genetic loci on chromosomes based on recombination frequencies, while a physical map is constructed based on overlapping restriction patterns of large insert BAC clones. Therefore, integration of these two different types of maps may reveal recombination hotspots as well as regions suppressed for recombination. In addition, this may allow an estimation of physical distances between genetic markers, thereby providing a framework for assembling the whole genome shotgun sequences.

Keeping in view the importance of integrating physical maps with genetic maps, we tried to anchor our physical map assembly with the genetic map by fingerprinting of 337 BAC clones and assembly of 319 of these clones into physical map assembly. The SSR markers derived from BAC-end sequencing of these clones had been found to be polymorphic and were mapped in the three bi-parental mapping populations derived from crosses ICC 4958 × PI 489777, ICC 4958 × ICC 1882 and ICC 283 × ICC 8261. Therefore, integrating these BAC clones into the physical map assembly helped to anchor our physical map with these three genetic maps (Supplementary Table 1).

The integration of physical map with genetic maps has been reported earlier in different model plant species including *Arabidopsis* (Meinke et al. 2009), rice (Chen et al. 2002) and fruit trees including peach (Zhebentyayeva et al. 2008), papaya (Yu et al. 2009) and apple (Han et al. 2011). The anchoring of physical map with the genetic map helped us to identify the BAC clones within or close to some of the QTLs already reported for important traits in chickpea (Supplementary Table 1). Therefore, the chickpea physical map may greatly help in cloning and fine mapping of some of these important genes in the future, thereby facilitating molecular breeding in this important crop.

Further, additional efforts are necessary to integrate and correlate the physical contig map with the other available genetic maps of chickpea for locating all mapped genes and QTLs to physical map contigs. These efforts have been further accelerated with the recent availability of the whole genome sequences of chickpea from NIPGR, New Delhi, India (Jain et al. 2013), and by the International Chickpea Genome Sequencing Consortium (Varshney et al. 2013b).

Integrated physical and genetic map is expected to act as an important resource for expediting the mapping and cloning of target genes controlling complex quantitative traits like drought and yield in chickpea. The availability of gene information will facilitate molecular breeding for improving important targeted traits in this important legume crop.

### Integrating physical map with the *Kabuli* chickpea genome map

Genome sequencing of all the model legumes including *Lotus* (Sato et al. 2008), soybean (Schmutz et al. 2010) and *Medicago* (Young et al. 2011) has been completed. Following model genomes, several other legume genomes have been sequenced including pigeon pea (Varshney et al. 2012), common bean (Scott Jackson, personal communications) and, recently, chickpea (Varshney et al. 2013b), and efforts are being made to sequence peanut, lentil and cowpea. This revolution in whole genome sequencing of crop genomes has made the integration of physical maps with the draft genome sequence possible. Chromosome-anchored physical maps may serve as an important tool for (i) improving the draft genome sequence by filling sequence gaps; (ii) resolving assembly errors caused due to repetitive sequences, large gene families and segmental duplications; (iii) providing anchor pints; (iv) ordering of sequence contigs/scaffolds; (v) map-based cloning; and (vi) cloning sequences that are too large or repetitive for PCR-based cloning (Ha et al. 2012).

Mapping of BESs from one variety onto the reference genome sequence of a different variety may help to identify putative chromosomal structural rearrangements between the two varieties. This has been done successfully for the identification of structural rearrangements between *Glycine soja* and *Glycine max* by aligning BESs from *G. soja* against the *G. max* reference sequence (Ha et al. 2012). Thus, physical maps will also help to investigate a structural evolution that has occurred between two genomes and to allow researchers to effectively shuttle between the genomes to capture useful information for crop improvement and basic genetics research (Ha et al. 2012).

The availability of genome sequence of CDC Frontier, a Canadian *Kabuli* chickpea variety (Varshney et al. 2013b), has facilitated the integration of BESs from the physical map including BES of MTP clones form the chickpea cultivar ICC 4958 with the daft sequence of chickpea. A set of only ~965 BACs including 163 MTP clones could be mapped onto the draft genome of chickpea. These BACs formed 491 hypothetical contigs representing 54,013,992 bp (~54 Mb) of reference genome. The mapping of 163 MTP clones on eight chickpea pseudo-molecule ranged from 11 (on Ca8) to 29 (Ca4) with an average of 20 clones/pseudo-molecule (Table 5). These mapped MTP clones coming from different contigs of the physical map represent ~70 Mb genome of physical map integrated into draft genome and genetic map. The low number of BACs mapped on the reference genome during the present study may be due to the following: (i) exclusion of 37,230 BES having different types of repeat elements; (ii) exclusion of BAC clones, where paired BESs aligned <100 and >200 kb apart; (iii) exclusion of majority of BACs showing multiple region mapping on either the same pseudo-molecule or different pseudo-molecules; and (iv) exclusion of several paired BES from mapping showing orientation in either the same or opposite directions indicating potential inversions. Similar type of potential inversions, repeat elements, mapping on different regions on pseudo-molecules have been reported earlier during the in silico mapping and integration of physical maps with the genome sequence in rice (Katagiri et al. 2004), tomato (Asamizu et al. 2012) and soybean (Ha et al. 2012). The removal of 37,230 repeat BES from chromosomal mapping was aimed to avoid the complications of map construction. Integration of the physical map of a *Desi* variety during the present study with the draft genome sequence of *Kabuli* chickpea will help in locating chromosomal structural arrangements between *Desi* (ICC 4958) and *Kabuli* (CDS Frontier) genotypes in future studies.

### Candidate stress-responsive genes

Among 654 genes present in the “*QTL-hotspot*”, the GO annotation-indicated genes like ERECTA-like kinase have a role in drought tolerance, and it has been reported that this gene enhances transpiration efficiency (Masle et al. 2005) and water use efficiency and transpiration efficiency in *Arabidopsis* (Xing et al. 2011). Several other genes like thiamine thiazole synthase and cysteine-rich receptor-like protein kinase, calmodulin-binding heat shock protein as well as other heat shock transcription factors, tubulin beta-chain, have roles in adaptation to various stress conditions. In addition, genes like fructose-bisphosphate aldolase, transmembrane protein which respond to salt stress as well as CRT/DRE-binding factor 4 gene which has shown a prominent role in abiotic stress response in *Medicago* (Li et al. 2011) were also found in this region, indicating the potential of this region to enhance the tolerance to several abiotic stresses.

In the case of *Ascochyta* blight resistance QTL region, among 306 genes, genes like BED finger-nbs resistance protein and gene with leucine-rich repeat domain are typically involved in host resistance mechanism like DNA-directed RNA polymerase subunit beta, receptor-like protein kinase and Ser-Thr protein kinase. Further, this region also harbours NAC domain protein for systemic acquired resistance as well as NB-LRR-type disease resistance protein Rps1-k-2 and ABC-type transport system involved in resistance to organic solvents’ periplasmic component.

Similarly, in the case of *Fusarium* wilt resistance QTL region, genes that have shown their activity in defense response to bacterium in *Medicago* like pheophorbide A oxygenase were found. This region also harbours superoxide dismutase which has shown differential expression in WR 315 compared to JG 62 in infected state as well as zinc finger protein and translation initiation factor (Gupta et al. 2013).

These candidate genes may be validated by using some functional genomics approaches like Targeting Induced Local Lesions IN Genome (TILLING), quantitative RT-PCR, over expression, etc., using the appropriate genetic material. In summary, integration of the physical map with the genetic maps and genome sequence of chickpea should help the international chickpea community for cloning of genes related to important tolerance/resistance to abiotic and biotic stresses as well as agronomic traits.

## Electronic supplementary material

Below is the link to the electronic supplementary material.

Supplementary Fig. 1 Comparison of sequence, physical and genetic maps. a-h are comparisons of each linkage group with respective psuedomolecules

High Resolution Image (TIFF 1145 kb)

High Resolution Image (TIFF 1015 kb)

High Resolution Image (TIFF 851 kb)

High Resolution Image (TIFF 984 kb)

High Resolution Image (TIFF 1520 kb)

High Resolution Image (TIFF 1219 kb)

High Resolution Image (TIFF 1028 kb)

High Resolution Image (TIFF 669 kb)

Supplementary Table 1 Markers mapped on to physical map and associated trait QTLs (DOCX 85 kb)

Supplementary Table 2 The distribution of repetitive sequences in 5,3316 BES used for their mapping on chickpea genome sequence (DOCX 17 kb)

Supplementary Table 3 List of genes in drought tolerance "*QTL-hotspot*" region (XLSX 46 kb)

Supplementary Table 4 List of genes in *Ascochyta* blight resistance QTL region (XLSX 30 kb)

Supplementary Table 5 List of genes in *Fusarium* wilt resistance QTL region (XLSX 12 kb)
