# Supplementary figures and images for: Integrated physical, genetic and genome map of chickpea (Cicer arietinum L.)
Source: Funct Integr Genomics. 2014 Mar 8;14(1):59–73. doi: 10.1007/s10142-014-0363-6 (PMC4273598; doi:10.1007/s10142-014-0363-6)

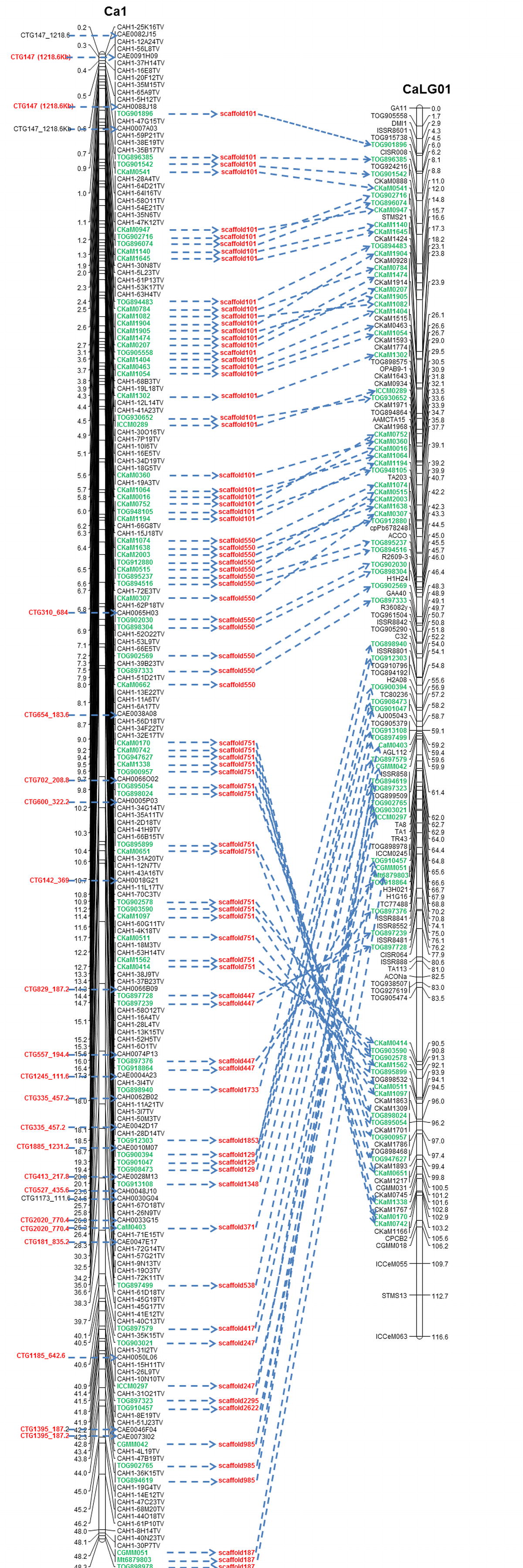

Supplement: Supplementary Fig. 1 — Comparison of sequence, physical and genetic maps. a-h are comparisons of each linkage group with respective psuedomolecules [file 10142_2014_363_Fig7_ESM.jpg]

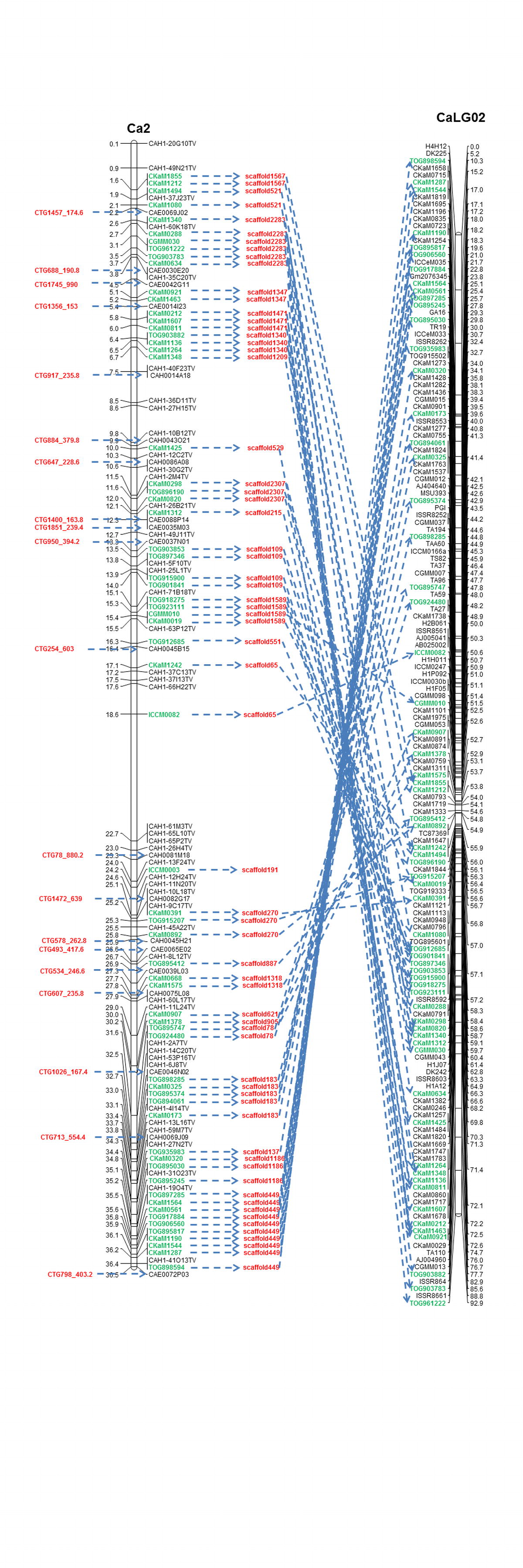

Supplement: Supplementary Fig. 1 — Comparison of sequence, physical and genetic maps. a-h are comparisons of each linkage group with respective psuedomolecules [file 10142_2014_363_Fig8_ESM.jpg]

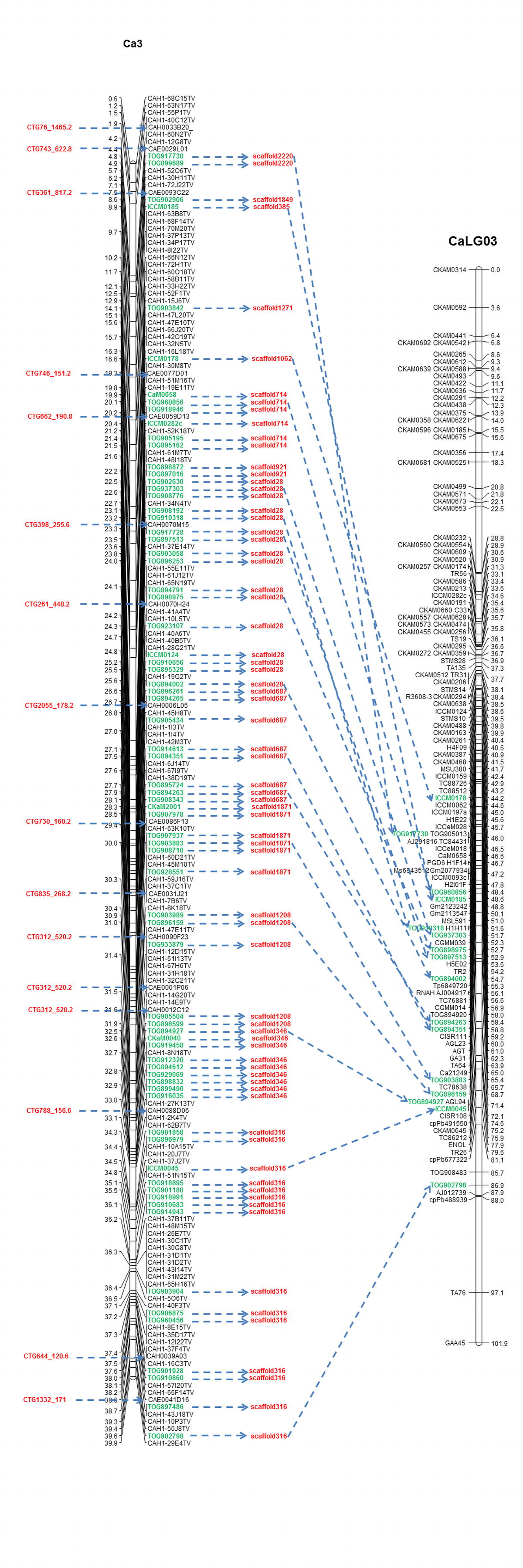

Supplement: Supplementary Fig. 1 — Comparison of sequence, physical and genetic maps. a-h are comparisons of each linkage group with respective psuedomolecules [file 10142_2014_363_Fig9_ESM.jpg]

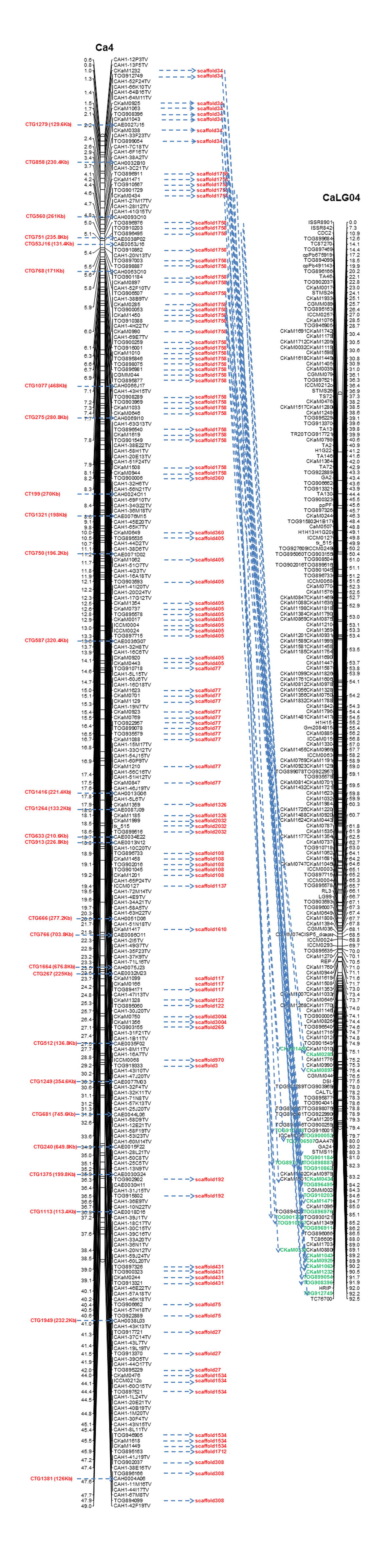

Supplement: Supplementary Fig. 1 — Comparison of sequence, physical and genetic maps. a-h are comparisons of each linkage group with respective psuedomolecules [file 10142_2014_363_Fig10_ESM.jpg]

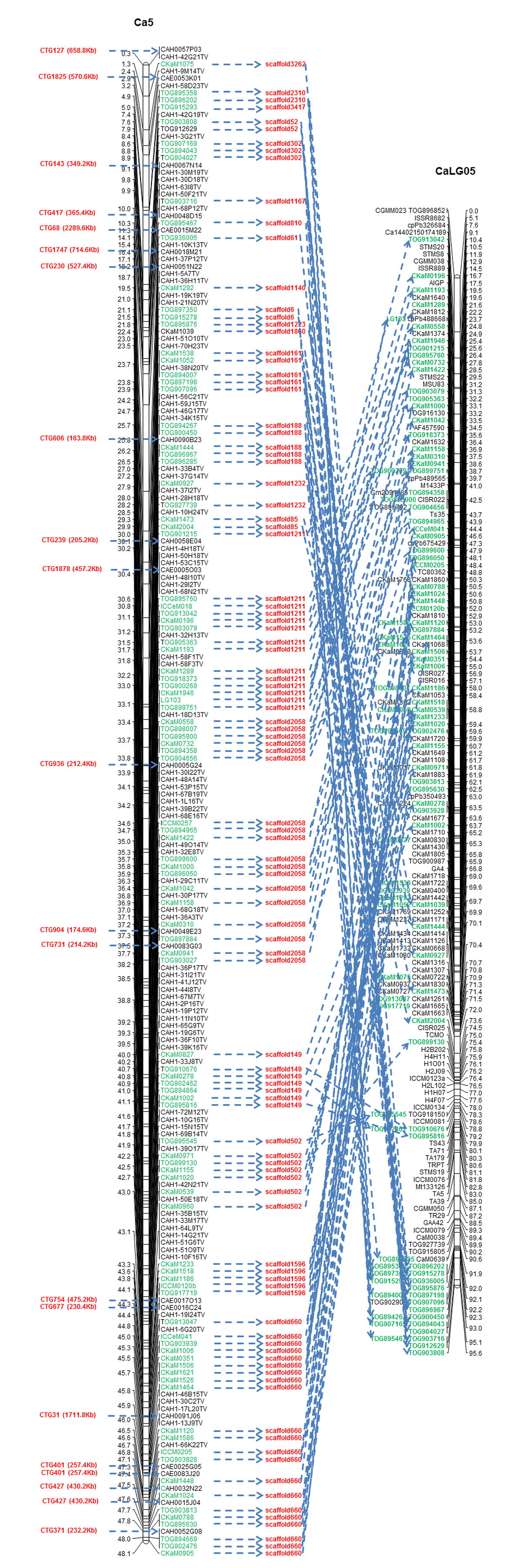

Supplement: Supplementary Fig. 1 — Comparison of sequence, physical and genetic maps. a-h are comparisons of each linkage group with respective psuedomolecules [file 10142_2014_363_Fig11_ESM.jpg]

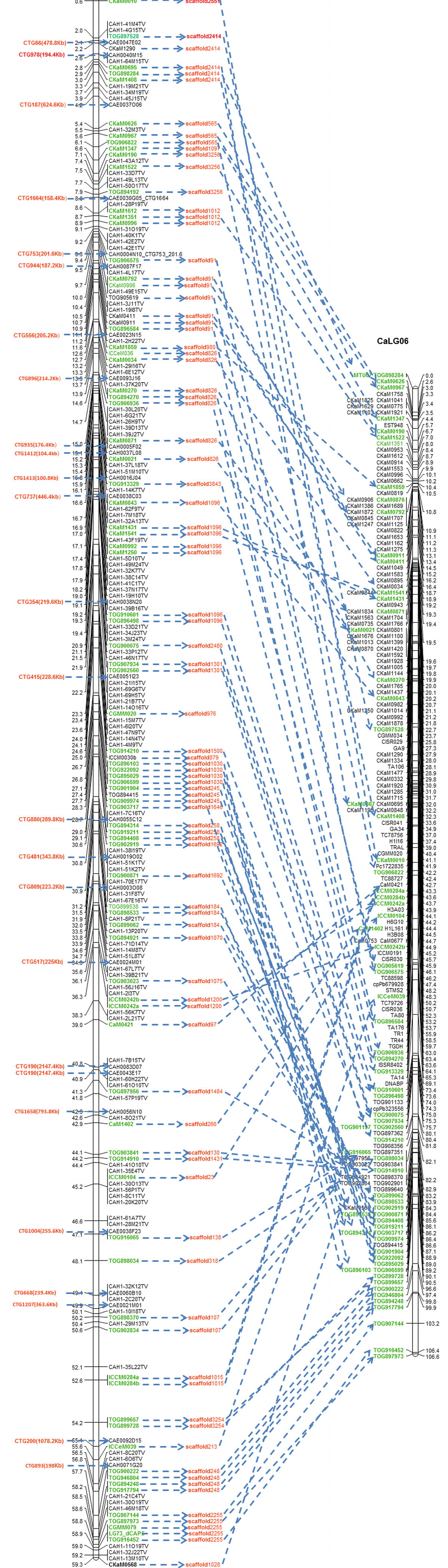

Supplement: Supplementary Fig. 1 — Comparison of sequence, physical and genetic maps. a-h are comparisons of each linkage group with respective psuedomolecules [file 10142_2014_363_Fig12_ESM.jpg]

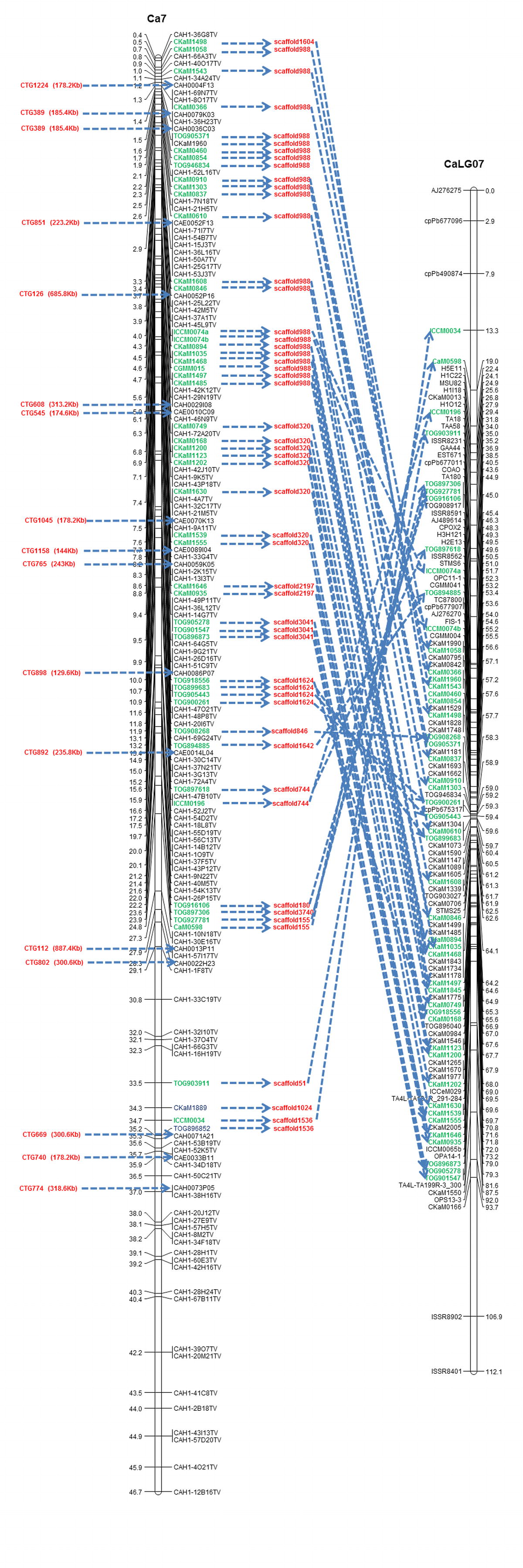

Supplement: Supplementary Fig. 1 — Comparison of sequence, physical and genetic maps. a-h are comparisons of each linkage group with respective psuedomolecules [file 10142_2014_363_Fig13_ESM.jpg]

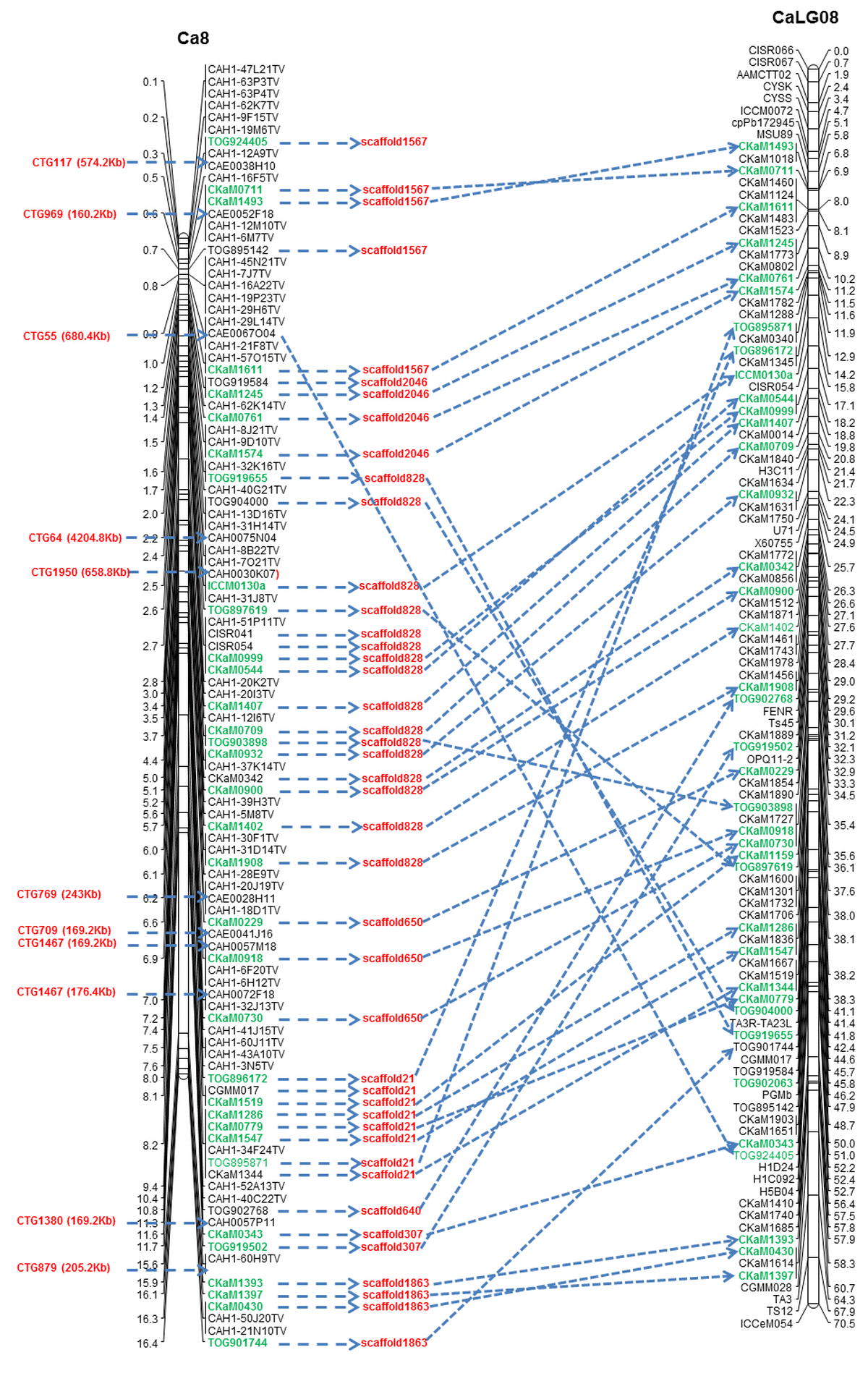

Supplement: Supplementary Fig. 1 — Comparison of sequence, physical and genetic maps. a-h are comparisons of each linkage group with respective psuedomolecules [file 10142_2014_363_Fig14_ESM.jpg]

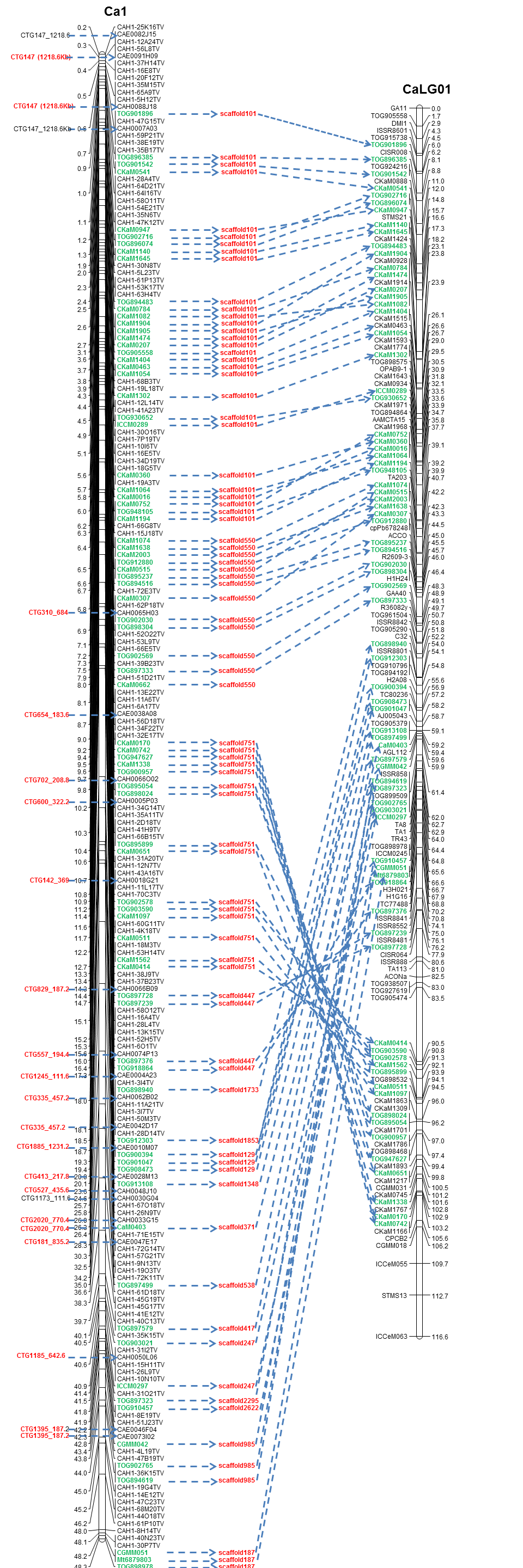

Supplement: Supplementary file 9 — High Resolution Image (TIFF 1145 kb) [file 10142_2014_363_MOESM1_ESM.tif]

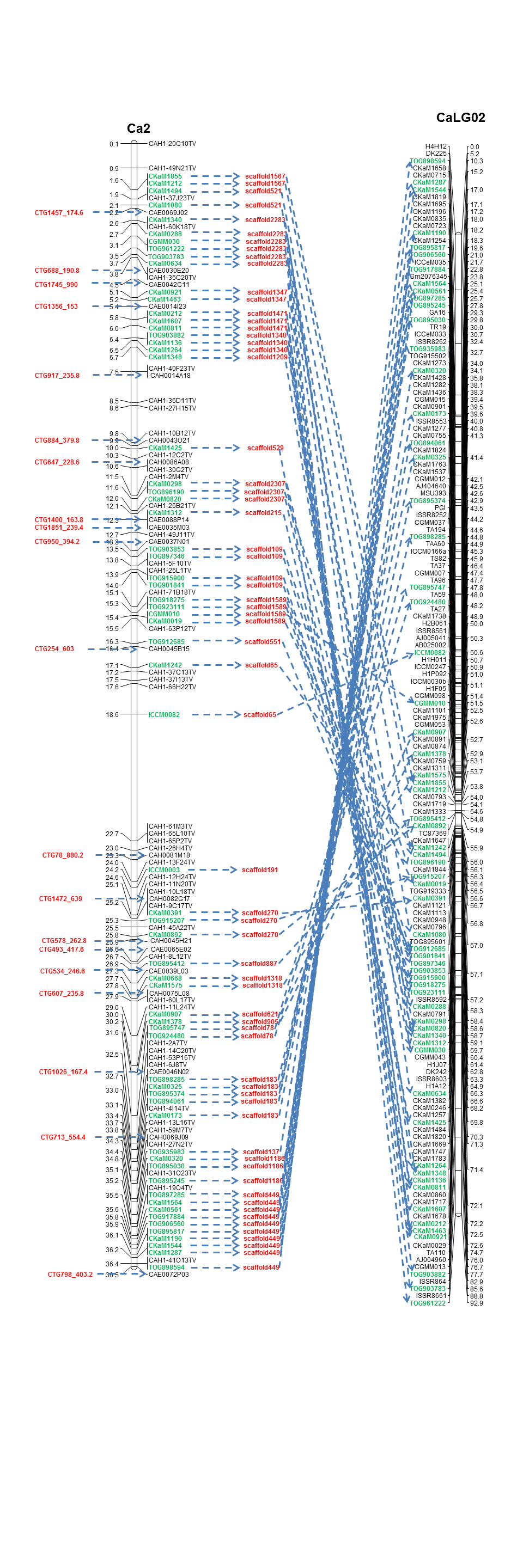

Supplement: Supplementary file 10 — High Resolution Image (TIFF 1015 kb) [file 10142_2014_363_MOESM2_ESM.tif]

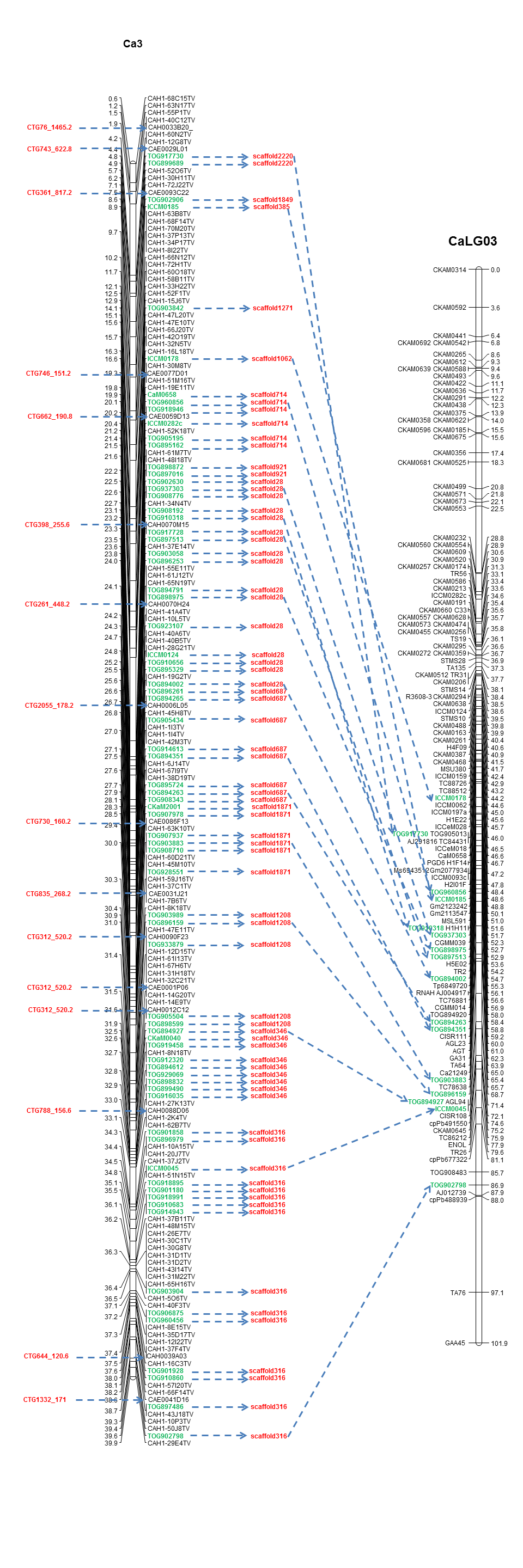

Supplement: Supplementary file 11 — High Resolution Image (TIFF 851 kb) [file 10142_2014_363_MOESM3_ESM.tif]

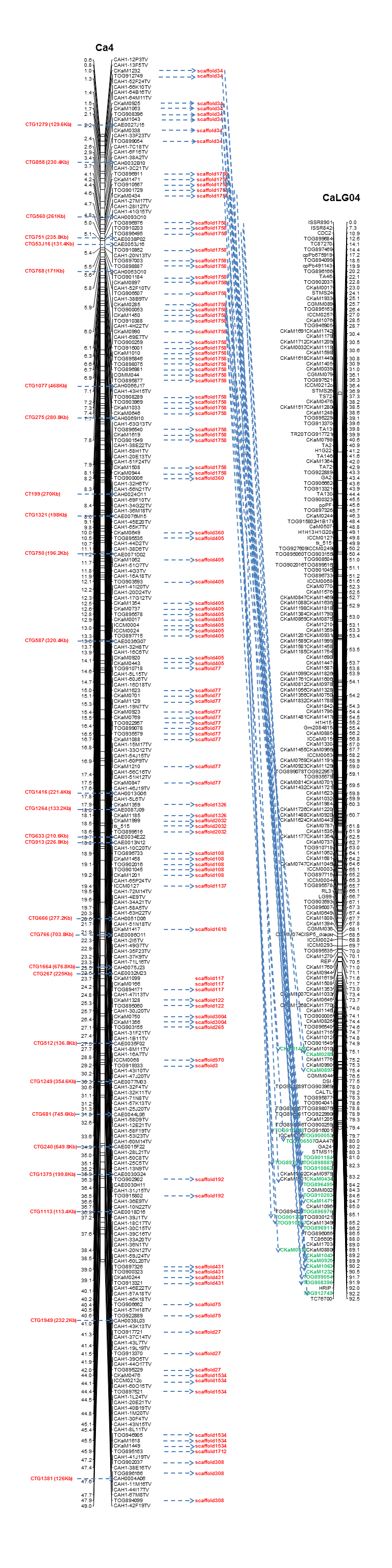

Supplement: Supplementary file 12 — High Resolution Image (TIFF 984 kb) [file 10142_2014_363_MOESM4_ESM.tif]

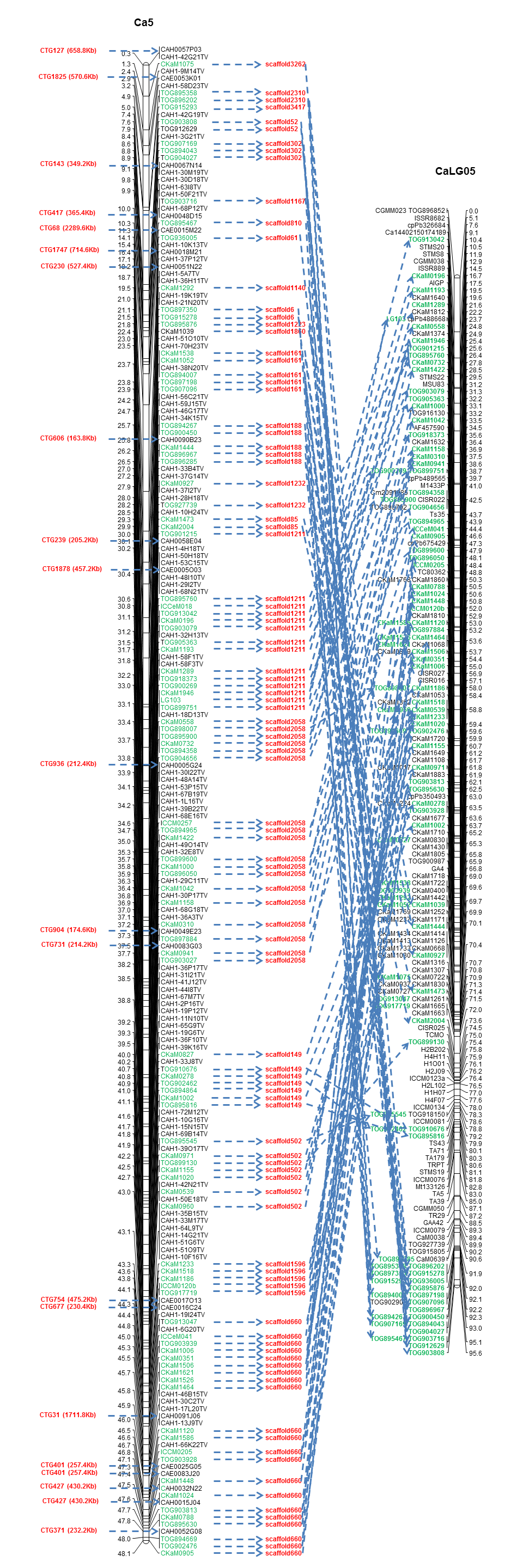

Supplement: Supplementary file 13 — High Resolution Image (TIFF 1520 kb) [file 10142_2014_363_MOESM5_ESM.tif]

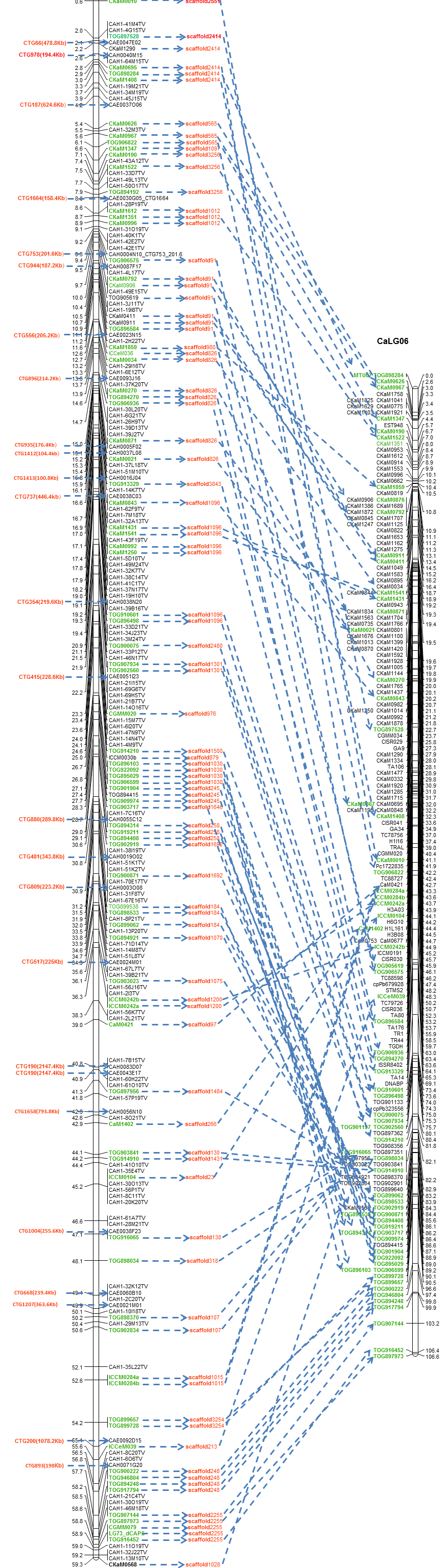

Supplement: Supplementary file 14 — High Resolution Image (TIFF 1219 kb) [file 10142_2014_363_MOESM6_ESM.tif]

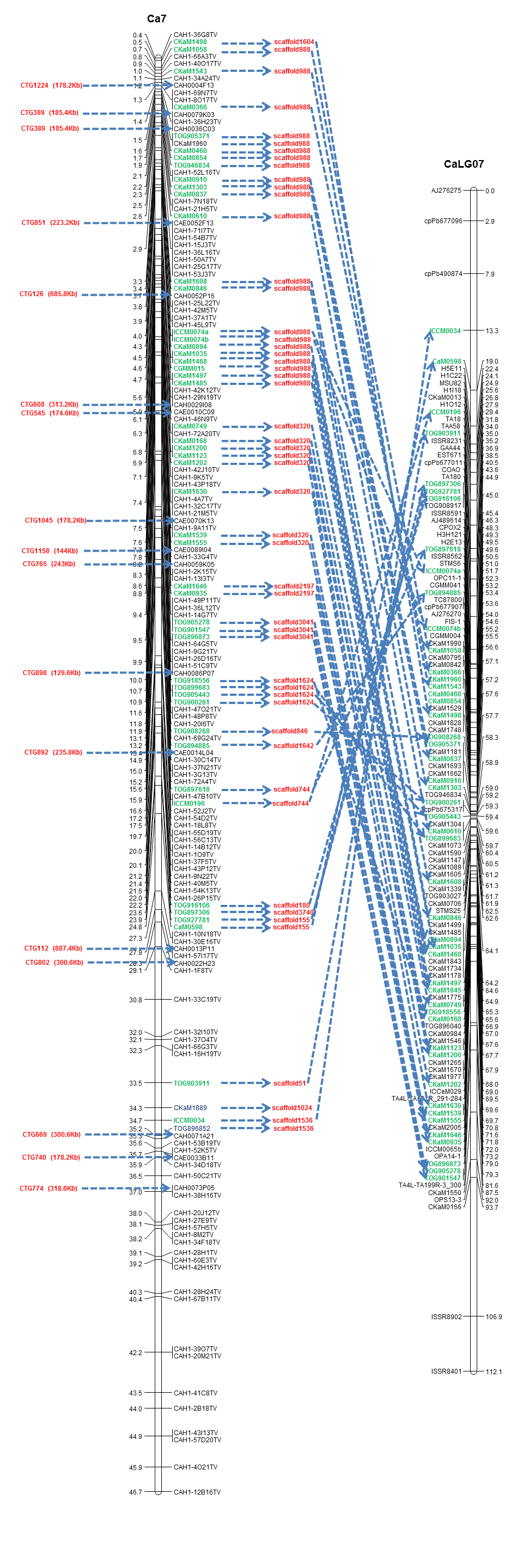

Supplement: Supplementary file 15 — High Resolution Image (TIFF 1028 kb) [file 10142_2014_363_MOESM7_ESM.tif]

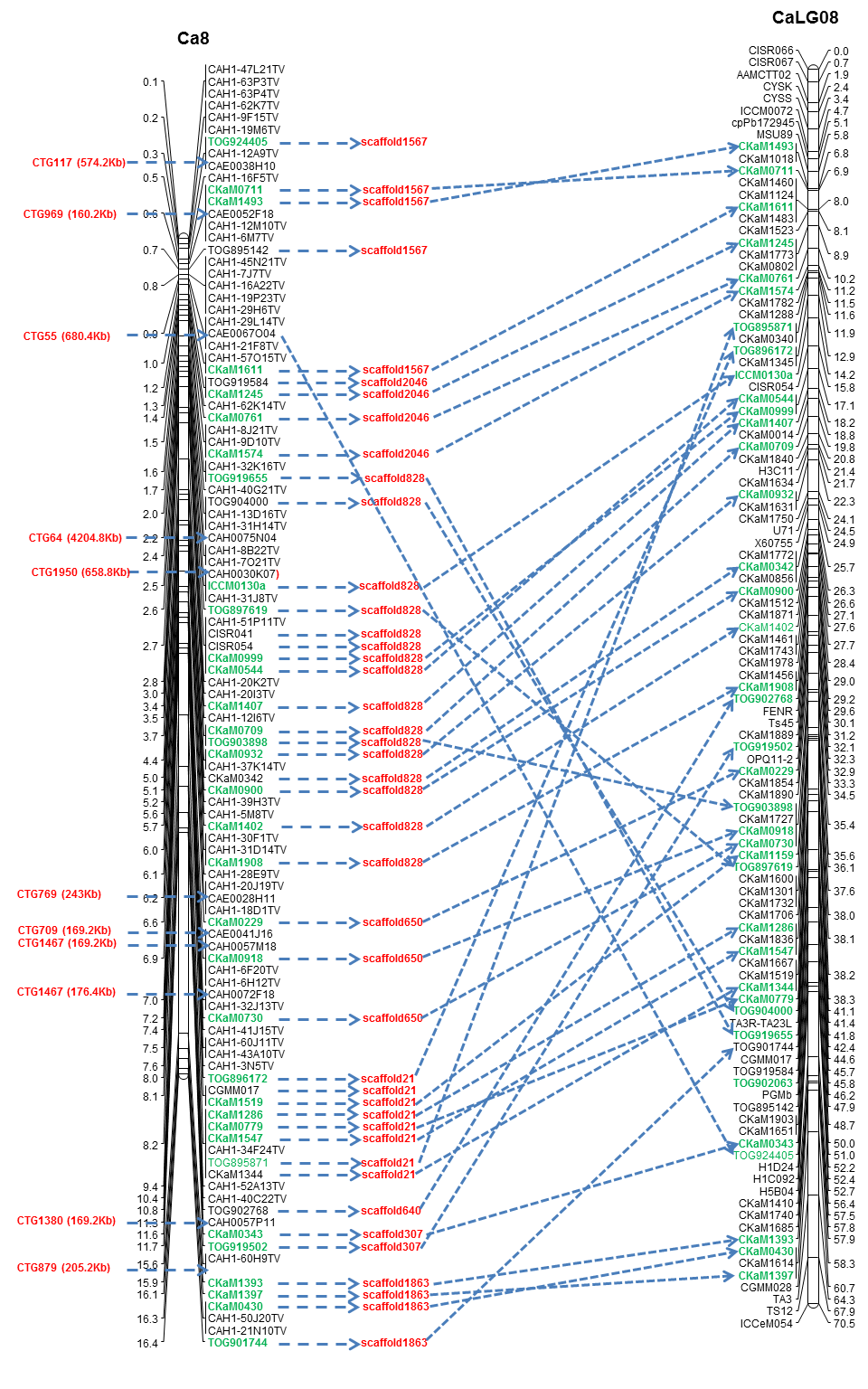

Supplement: Supplementary file 16 — High Resolution Image (TIFF 669 kb) [file 10142_2014_363_MOESM8_ESM.tif]
