# Supplementary material for: Integrated physical, genetic and genome map of chickpea (Cicer arietinum L.)
Source: Funct Integr Genomics. 2014 Mar 8;14(1):59–73. doi: 10.1007/s10142-014-0363-6 (PMC4273598; doi:10.1007/s10142-014-0363-6)
Supplement: Supplementary file 17 — Markers mapped on to physical map and associated trait QTLs (DOCX 85 kb) [file 10142_2014_363_MOESM9_ESM.docx]

**Supplementary Table 1: Markers mapped on to physical map and associated trait QTLs**

| **Marker** | | **Genbank ID** | | **Position on genetic map (Linkage group; distance in cM)** | | **BAC clone ID** | | **Contig ID** | | **No of clones/ contig** | | **Size (kb)** | | **QTL information (Trait; Phenotypic variation %)** | | **Reference** | |
| --- | --- | --- | --- | --- | --- | --- | --- | --- | --- | --- | --- | --- | --- | --- | --- | --- | --- |
| CaM0003 | | EI846602 | | CaLG04; 29.46 | | CAH1001G03 | | ctg45 | | 348 | | 45240 | |  | | Thudi et al. (2011) | |
| CaM0015 | | EI846874 | | CaLG04; 29.46 | | CAH1001F17 | | ctg206 | | 59 | | 7670 | | Biomass (5.62) | | Varshney et al. 20142014 | |
| CaM0034 | | EI847407 | | CaLG07; 53.05 | | CAH1006F19 | | ctg664 | | 10 | | 1300 | |  | | Thudi et al. (2011) | |
| CaM0038 | | EI847480 | | CaLG05; 99.97 | | CAH10017D16 | | ctg697 | | 102 | | 13260 | |  | | Thudi et al. (2011) | |
| CaM0043 | | EI847726 | | CaLG05; 99.97 | | CAH1003A09 | | ctg169 | | 287 | | 37310 | |  | | Thudi et al. (2011) | |
| CaM0046 | | EI847762 | | CaLG05; 99.97 | | CAH1003G09 | | ctg96 | | 36 | | 4680 | | DTI (5.15-11.23), Yield (13.97) | | Varshney et al. 2014 | |
| CaM0051 | | EI847897 | | CaLG05; 99.97 | | CAH1005O07 | | ctg673 | | 675 | | 87750 | |  | | Thudi et al. (2011) | |
| CaM0063 | | EI848286 | | CaLG06; 77.10 | | CAH1007C16 | | ctg0 | | - | | - | |  | | Thudi et al. (2011) | |
| CaM0081 | | EI848879 | | CaLG06; 77.10 | | CAH1007P16 | | ctg1885 | | 154 | | 20020 | |  | | Thudi et al. (2011) | |
| CaM0102 | | EI849280 | | CaLG06; 77.10 | | CAH1013J07 | | ctg1994 | | 133 | | 17290 | |  | | Thudi et al. (2011) | |
| CaM0111 | | EI849398 | | CaLG06; 77.10 | | CAH1018O04 | | ctg112 | | 76 | | 9880 | |  | | Thudi et al. (2011) | |
| CaM0113 | | EI849479 | | CaLG04; 61.45 | | CAH1012N03 | | ctg95 | | 107 | | 13910 | |  | | Thudi et al. (2011) | |
| CaM0120 | | EI849587 | | CaLG04; 61.45 | | CAH1018B01 | | ctg51 | | 107 | | 13910 | |  | | Thudi et al. (2011) | |
| CaM0123 | | EI849604 | | CaLG05; 21.41 | | CAH1018D13 | | ctg1263 | | 3 | | 390 | |  | | Thudi et al. (2011) | |
| CaM0142 | | EI849925 | | CaLG05; 21.41 | | CAH1009M19 | | ctg447 | | 146 | | 18980 | |  | | Thudi et al. (2011) | |
| CaM0144 | | EI849960 | | CaLG05; 21.41 | | CAH1018C19 | | ctg0 | | - | | - | | Biomass (5.62) | | Varshney et al. 2014 | |
| CaM0157 | | EI850153 | | CaLG05; 21.41 | | CAH1018G18 | | ctg80 | | 88 | | 11440 | |  | | Thudi et al. (2011) | |
| CaM0169 | | EI850512 | | CaLG05; 21.41 | | CAH1018H06 | | ctg1150 | | 8 | | 1040 | |  | | Thudi et al. (2011) | |
| CaM0173 | | EI850588 | | CaLG06; 12.82 | | CAH1020E05 | | ctg1177 | | 24 | | 3120 | |  | | Thudi et al. (2011) | |
| CaM0204 | | EI851302 | | CaLG06; 12.82 | | CAH1020D19 | | ctg76 | | 116 | | 15080 | |  | | Thudi et al. (2011) | |
| **Marker** | | **Genbank ID** | | **Position on genetic map (Linkage group; distance in cM)** | | **BAC clone ID** | | **Contig ID** | | **No of clones/ contig** | | **Size (kb)** | | **QTL information (Trait; Phenotypic variation %)** | | **Reference** | |
| CaM0232 | | EI852150 | | CaLG04; 56.66 | | CAH1015M17 | | ctg198 | | 33 | | 4290 | | Seed weight QTL1 (on LG4B) and drought (on LG4) | | Abbo et al. (2005) | |
| CaM0233 | | EI852173 | | CaLG06; 11.40 | | CAH1009A20 | | ctg1755 | | 113 | | 14690 | |  | | Thudi et al. (2011) | |
| CaM0244 | | EI852407 | | CaLG06; 56.23 | | CAH1009L15 | | ctg567 | | 32 | | 4160 | |  | | Thudi et al. (2011) | |
| CaM0251 | | EI852496 | | CaLG06; 78.21 | | CAH1015L17 | | ctg450 | | 25 | | 3250 | |  | | Thudi et al. (2011) | |
| CaM0258 | | EI852603 | | CaLG05; 89.82 | | CAH1009N24 | | ctg248 | | 36 | | 4680 | |  | | Thudi et al. (2011) | |
| CaM0260 | | EI852656 | | CaLG03; 16.41 | | CAH1015J06 | | ctg27 | | 288 | | 37440 | |  | | Thudi et al. (2011) | |
| CaM0277 | | EI852999 | | CaLG07; 27.41 | | CAH1020I06 | | ctg1177 | | 24 | | 3120 | |  | | Thudi et al. (2011) | |
| CaM0286 | | EI853206 | | CaLG07; 50.58 | | CAH1011O16 | | ctg266 | | 47 | | 6110 | |  | | Thudi et al. (2011) | |
| CaM0293 | | EI853320 | | CaLG07; 50.58 | | CAH1013D19 | | ctg7 | | 500 | | 65000 | |  | | Thudi et al. (2011) | |
| CaM0317 | | EI854030 | | CaLG07; 50.58 | | CAH1015I17 | | ctg237 | | 26 | | 3380 | |  | | Thudi et al. (2011) | |
| CaM0326 | | EI854206 | | CaLG07; 50.58 | | CAH1003K08 | | ctg95 | | 107 | | 13910 | |  | | Thudi et al. (2011) | |
| CaM0336 | | EI854339 | | CaLG02; 53.76 | | CAH1021C12 | | ctg185 | | 82 | | 10660 | | AB, FW | | Varshney et al. 2014 | |
| CaM0340 | | EI854366 | | CaLG07; 31.08 | | CAH1021G22 | | ctg36 | | 144 | | 18720 | |  | | Thudi et al. (2011) | |
| CaM0345 | | EI854527 | | CaLG07; 31.52 | | CAH1014E14 | | ctg15 | | 240 | | 31200 | |  | | Thudi et al. (2011) | |
| CaM0358 | | EI854746 | | CaLG05; 89.38 | | CAH1003P15 | | ctg1776 | | 260 | | 33800 | |  | | Thudi et al. (2011) | |
| CaM0368 | | EI854954 | | CaLG05; 97.41 | | CAH1014D15 | | ctg59 | | 72 | | 9360 | |  | | Thudi et al. (2011) | |
| CaM0389 | | EI855460 | | CaLG05; 97.41 | | CAH1014C05 | | ctg1768 | | 64 | | 8320 | |  | | Thudi et al. (2011) | |
| CaM0391 | | EI855489 | | CaLG05; 97.41 | | CAH1014G19 | | ctg245 | | 38 | | 4940 | |  | | Thudi et al. (2011) | |
| CaM0393 | | EI855520 | | CaLG05; 97.41 | | CAH1014M11 | | ctg181 | | 65 | | 8450 | | Harvest index (14.35) | | Varshney et al. 2014 | |
| **Marker** | | **Genbank ID** | | **Position on genetic map (Linkage group; distance in cM)** | | **BAC clone ID** | | **Contig ID** | | **No of clones/ contig** | | **Size (kb)** | | **QTL information (Trait; Phenotypic variation %)** | | **Reference** | |
| CaM0399 | | EI855580 | | CaLG05; 97.41 | | CAH1004G15 | | ctg1778 | | 62 | | 8060 | | Plant height (8.42); Days to maturity (12.13) | | Thudi et al. (2011) | |
| CaM0403 | | EI855604 | | CaLG01; 47.97 | | CAH1004K15 | | ctg206 | | 59 | | 7670 | |  | | Thudi et al. (2011) | |
| CaM0416 | | EI855812 | | CaLG05; 99.91 | | CAH1014O18 | | ctg16 | | 293 | | 38090 | |  | | Thudi et al. (2011) | |
| CaM0421 | | EI855939 | | CaLG06; 79.03 | | CAH1002E18 | | ctg104 | | 77 | | 10010 | |  | | Thudi et al. (2011) | |
| CaM0423 | | EI856010 | | CaLG05; 100.45 | | CAH1004B23 | | ctg1127 | | 122 | | 15860 | |  | | Thudi et al. (2011) | |
| CaM0435 | | EI856279 | | CaLG07; 32.60 | | CAH1014D06 | | ctg699 | | 8 | | 1040 | |  | | Thudi et al. (2011) | |
| CaM0436 | | EI856290 | | CaLG04; 63.63 | | CAH1014F08 | | ctg44 | | 217 | | 28210 | |  | | Thudi et al. (2011) | |
| CaM0443 | | EI856442 | | CaLG07; 37.99 | | CAH1004P16 | | ctg252 | | 3007 | | 390910 | |  | | Thudi et al. (2011) | |
| CaM0446 | | EI856503 | | CaLG04; 61.74 | | CAH1002L12 | | ctg525 | | 9 | | 1170 | |  | | Thudi et al. (2011) | |
| CaM0463 | | EI856964 | | CaLG05; 61.02 | | CAH1002K24 | | ctg642 | | 7 | | 910 | |  | | Thudi et al. (2011) | |
| CaM0464 | | EI856986 | | CaLG06; 70.09 | | CAH1002O22 | | ctg1865 | | 9 | | 1170 | |  | | Thudi et al. (2011) | |
| CaM0475 | | EI857186 | | CaLG03; 31.25 | | CAH1002F07 | | ctg61 | | 186 | | 24180 | | Beta carotene QTL2 (on LG3B) | | Abbo et al. (2005) | |
| CaM0480 | | EI857283 | | CaLG04; 61.60 | | CAH1006H13 | | ctg95 | | 107 | | 13910 | |  | | Thudi et al. (2011) | |
| CaM0486 | | EI857434 | | CaLG01; 48.40 | | CAH1002F04 | | ctg434 | | 78 | | 10140 | |  | | Thudi et al. (2011) | |
| CaM0491 | | EI857496 | | CaLG05; 108.76 | | CAH1002P16 | | ctg725 | | 7 | | 910 | |  | | Thudi et al. (2011) | |
| CaM0492 | | EI857532 | | CaLG08; 62.74 | | CAH1006F20 | | ctg664 | | 10 | | 1300 | |  | | Thudi et al. (2011) | |
| CaM0493 | | EI857539 | | CaLG08; 60.61 | | CAH1006H12 | | ctg709 | | 6 | | 780 | |  | | Thudi et al. (2011) | |
| CaM0499 | | EI857748 | | CaLG08; 60.61 | | CAH1008O05 | | ctg77 | | 39 | | 5070 | |  | | Thudi et al. (2011) | |
| CaM0500 | | EI857756 | | CaLG08; 60.61 | | CAH1008O21 | | ctg1658 | | 75 | | 9750 | |  | | Thudi et al. (2011) | |
| CaM0507 | | EI857918 | | CaLG04; 60.91 | | CAH1016M21 | | ctg58 | | 48 | | 6240 | |  | | Thudi et al. (2011) | |
| CaM0519 | | EI858190 | | CaLG03; 11.43 | | CAH1016A14 | | ctg536 | | 14 | | 1820 | |  | | Thudi et al. (2011) | |
| **Marker** | | **Genbank ID** | | **Position on genetic map (Linkage group; distance in cM)** | | **BAC clone ID** | | **Contig ID** | | **No of clones/ contig** | | **Size (kb)** | | **QTL information (Trait; Phenotypic variation %)** | | **Reference** | |
| CaM0520 | | EI858211 | | CaLG03; 11.43 | | CAH1016E14 | | ctg0 | | - | | - | |  | | Thudi et al. (2011) | |
| CaM0539 | | EI858666 | | CaLG08; 54.82 | | CAH1007L17 | | ctg153 | | 284 | | 36920 | |  | | Thudi et al. (2011) | |
| CaM0574 | | EI860034 | | CaLG05; 100.70 | | CAH1017K18 | | ctg1320 | | 34 | | 4420 | |  | | Thudi et al. (2011) | |
| CaM0583 | | EI860226 | | CaLG07; 65.41 | | CAH1006O06 | | ctg889 | | 5 | | 650 | |  | | Thudi et al. (2011) | |
| CaM0594 | | EI860404 | | CaLG06; 65.65 | | CAH1010O12 | | ctg635 | | 10 | | 1300 | |  | | Thudi et al. (2011) | |
| CaM0598 | | EI860454 | | CaLG07; 47.98 | | CAH1008G22 | | ctg57 | | 105 | | 13650 | |  | | Thudi et al. (2011) | |
| CaM0599 | | EI860464 | | CaLG07; 31.84 | | CAH1008I20 | | ctg1369 | | 9 | | 1170 | |  | | Thudi et al. (2011) | |
| CaM0600 | | EI860465 | | CaLG03; 19.33 | | CAH1008I22 | | ctg53 | | 74 | | 9620 | | Beta carotene QTL2 (on LG3B) | | Abbo et al. (2005) | |
| CaM0610 | | EI860570 | | CaLG03; 56.24 | | CAH108N18 | | ctg58 | | 48 | | 6240 | |  | | Thudi et al. (2011) | |
| CaM0615 | | EI860705 | | CaLG03; 56.24 | | CAH1005H05 | | ctg33 | | 392 | | 50960 | |  | | Thudi et al. (2011) | |
| CaM0620 | | EI860849 | | CaLG06; 78.32 | | CAH1011C01 | | ctg72 | | 186 | | 24180 | |  | | Thudi et al. (2011) | |
| CaM0622 | | EI860872 | | CaLG07; 50.11 | | CAH1011G01 | | ctg1931 | | 45 | | 5850 | |  | | Thudi et al. (2011) | |
| CaM0624 | | EI860886 | | CaLG03; 30.22 | | CAH1011I09 | | ctg33 | | 392 | | 50960 | | Beta carotene QTL2 (on LG3B) | | Abbo et al. (2005) | |
| CaM0629 | | EI860976 | | CaLG03; 30.64 | | CAH1011I10 | | ctg33 | | 392 | | 50960 | | Beta carotene QTL2 (on LG3B) | | Abbo et al. (2005) | |
| CaM0632 | | EI861006 | | CaLG03; 10.64 | | CAH1011M24 | | ctg233 | | 41 | | 5330 | |  | | Thudi et al. (2011) | |
| CaM0636 | | EI861078 | | CaLG03; 10.64 | | CAH1011L13 | | ctg567 | | 32 | | 4160 | |  | | Thudi et al. (2011) | |
| CaM0639 | | EI861153 | | CaLG05; 101.73 | | CAH1011J12 | | ctg1431 | | 3 | | 390 | |  | | Thudi et al. (2011) | |
| CaM0645 | | EI861227 | | CaLG04; 62.60 | | CAH1037G05 | | ctg365 | | 99 | | 12870 | |  | | Thudi et al. (2011) | |
| CaM0656 | | EI861395 | | CaLG07; 18.95 | | CAH1025E19 | | ctg1751 | | 123 | | 15990 | |  | | Thudi et al. (2011) | |
| **Marker** | | **Genbank ID** | | **Position on genetic map (Linkage group; distance in cM)** | | **BAC clone ID** | | **Contig ID** | | **No of clones/ contig** | | **Size (kb)** | | **QTL information (Trait; Phenotypic variation %)** | | **Reference** | |
| CaM0658 | | EI861468 | | CaLG03; 30.69 | | CAH1037C12 | | ctg111 | | 72 | | 9360 | | Beta carotene QTL2 (on LG3B) | | Abbo et al. (2005) | |
| CaM0661 | | EI861555 | | CaLG07; 53.99 | | CAH1045C12 | | ctg110 | | 106 | | 13780 | |  | | Thudi et al. (2011) | |
| CaM0677 | | EI861783 | | CaLG06; 78.64 | | CAH1037L21 | | ctg24 | | 123 | | 15990 | |  | | Thudi et al. (2011) | |
| CaM0691 | | EI862180 | | CaLG04; 67.40 | | CAH1025F16 | | ctg1755 | | 113 | | 14690 | |  | | Thudi et al. (2011) | |
| CaM0694 | | EI862295 | | CaLG04; 67.40 | | CAH1031M01 | | ctg523 | | 20 | | 2600 | | Biomass (6.3824) | | Varshney et al. 2014 | |
| CaM0698 | | EI862402 | | CaLG05; 93.56 | | CAH1031A12 | | ctg281 | | 34 | | 4420 | |  | | Thudi et al. (2011) | |
| CaM0705 | | EI862538 | | CaLG07; 28.94 | | CAH1026K08 | | ctg190 | | 492 | | 63960 | |  | | Thudi et al. (2011) | |
| CaM0713 | | EI862793 | | CaLG07; 28.94 | | CAH1031L22 | | ctg536 | | 14 | | 1820 | |  | | Thudi et al. (2011) | |
| CaM0717 | | EI862873 | | CaLG03; 30.79 | | CAH1026N04 | | ctg80 | | 88 | | 11440 | | Beta carotene QTL2 (on LG3B) | | Abbo et al. (2005) | |
| CaM0720 | | EI863044 | | CaLG01; 48.63 | | CAH1027O03 | | ctg458 | | 10 | | 1300 | |  | | Thudi et al. (2011) | |
| CaM0726 | | EI863145 | | CaLG06; 8.374 | | CAH1045A22 | | ctg408 | | 12 | | 1560 | |  | | Thudi et al. (2011) | |
| CaM0740 | | EI863370 | | CaLG05; 81.87 | | CAH1031M06 | | ctg1176 | | 6 | | 780 | |  | | Thudi et al. (2011) | |
| CaM0743 | | EI863386 | | CaLG06; 88.45 | | CAH1031O18 | | ctg198 | | 33 | | 4290 | |  | | Thudi et al. (2011) | |
| CaM0744 | | EI863387 | | CaLG06; 88.45 | | CAH1031O20 | | ctg0 | | - | | - | |  | | Thudi et al. (2011) | |
| CaM0751 | | EI863561 | | CaLG03; 10.82 | | CAH1031B21 | | ctg0 | | - | | - | |  | | Thudi et al. (2011) | |
| CaM0753 | | EI863632 | | CaLG06; 78.58 | | CAH1031P07 | | ctg27 | | 288 | | 37440 | |  | | Thudi et al. (2011) | |
| CaM0772 | | EI863992 | | CaLG06; 78.58 | | CAH1025G05 | | ctg55 | | 53 | | 6890 | |  | | Thudi et al. (2011) | |
| CaM0787 | | EI864268 | | CaLG08; 28.90 | | CAH1037K14 | | ctg0 | | - | | - | |  | | Thudi et al. (2011) | |
| CaM0790 | | EI864296 | | CaLG03; 9.37 | | CAH1027B01 | | ctg233 | | 41 | | 5330 | |  | | Thudi et al. (2011) | |
| CaM0795 | | EI864439 | | CaLG07; 30.23 | | CAH1025P01 | | ctg15 | | 240 | | 31200 | |  | | Thudi et al. (2011) | |
| **Marker** | | **Genbank ID** | | **Position on genetic map (Linkage group; distance in cM)** | | **BAC clone ID** | | **Contig ID** | | **No of clones/ contig** | | **Size (kb)** | | **QTL information (Trait; Phenotypic variation %)** | | **Reference** | |
| CaM0797 | | EI864564 | | CaLG01; 93.52 | | CAH1037H14 | | ctg147 | | 82 | | 10660 | | Beta carotene QTL3 and Seed weight QTL 2 (1B) | | Abbo et al. (2005) | |
| CaM0799 | | EI864569 | | CaLG03; 2.648 | | CAH1037J02 | | ctg0 | | - | | - | |  | | Thudi et al. (2011) | |
| CaM0800 | | EI864573 | | CaLG03; 2.648 | | CAH1037J12 | | ctg0 | | - | | - | |  | | Thudi et al. (2011) | |
| CaM0805 | | EI864627 | | CaLG05; 81.48 | | CAH1025D10 | | ctg17 | | 54 | | 7020 | |  | | Thudi et al. (2011) | |
| CaM0806 | | EI864628 | | CaLG06; 78.34 | | CAH1025D12 | | ctg104 | | 77 | | 10010 | |  | | Thudi et al. (2011) | |
| CaM0812 | | EI864718 | | CaLG07; 0 | | CAH1025L22 | | ctg126 | | 44 | | 5720 | |  | | Thudi et al. (2011) | |
| CaM0814 | | EI864799 | | CaLG08; 56.84 | | CAH1028E09 | | ctg769 | | 11 | | 1430 | |  | | Thudi et al. (2011) | |
| CaM0821 | | EI864888 | | CaLG08; 56.84 | | CAH1026E23 | | ctg13 | | 64 | | 8320 | |  | | Thudi et al. (2011) | |
| CaM0836 | | EI865537 | | CaLG05; 92.42 | | CAH1028F24 | | ctg673 | | 675 | | 87750 | |  | | Thudi et al. (2011) | |
| CaM0845 | | EI865873 | | CaLG05; 92.42 | | CAH1029E03 | | ctg33 | | 392 | | 50960 | |  | | Thudi et al. (2011) | |
| CaM0848 | | EI865930 | | CaLG05; 92.42 | | CAH1029O15 | | ctg419 | | 19 | | 2470 | |  | | Thudi et al. (2011) | |
| CaM0856 | | EI866026 | | CaLG05; 92.42 | | CAH1030A10 | | ctg240 | | 33 | | 4290 | |  | | Thudi et al. (2011) | |
| CaM0861 | | EI866100 | | CaLG05; 92.42 | | CAH1030M20 | | ctg12 | | 201 | | 26130 | |  | | Thudi et al. (2011) | |
| CaM0862 | | EI866127 | | CaLG03; 59.45 | | CAH1030C04 | | ctg700 | | 11 | | 1430 | |  | | Thudi et al. (2011) | |
| CaM0864 | | EI866131 | | CaLG07; 25.17 | | CAH1030C14 | | ctg469 | | 13 | | 1690 | |  | | Thudi et al. (2011) | |
| CaM0880 | | EI866471 | | CaLG05; 37.38 | | CAH1030P17 | | ctg0 | | - | | - | |  | | Thudi et al. (2011) | |
| CaM0881 | | EI866471 | | CaLG05; 36.93 | | CAH1030P17 | | ctg0 | | - | | - | |  | | Thudi et al. (2011) | |
| CaM0886 | | EI866534 | | CaLG03; 45.07 | | CAH1030L03 | | ctg42 | | 263 | | 34190 | | Beta carotene QTL2 (on LG3B) | | Abbo et al. (2005) | |
| CaM0899 | | EI866951 | | CaLG03; 45.07 | | CAH1029J08 | | ctg134 | | 100 | | 13000 | |  | | Thudi et al. (2011) | |
| CaM0906 | | EI867102 | | CaLG03; 45.07 | | CAH1036G07 | | ctg464 | | 10 | | 1300 | |  | | Thudi et al. (2011) | |
| **Marker** | | **Genbank ID** | | **Position on genetic map (Linkage group; distance in cM)** | | **BAC clone ID** | | **Contig ID** | | **No of clones/ contig** | | **Size (kb)** | | **QTL information (Trait; Phenotypic variation %)** | | **Reference** | |
| CaM0909 | | EI867184 | | CaLG04; 61.09 | | CAH1038E15 | | ctg33 | | 392 | | 50960 | |  | | Thudi et al. (2011) | |
| CaM0923 | | EI867368 | | CaLG04; 61.09 | | CAH1035E19 | | ctg337 | | 19 | | 2470 | | 100-seed weight (3.99) | | Varshney et al. 2014 | |
| CaM0924 | | EI867368 | | CaLG04; 61.09 | | CAH1035E19 | | ctg337 | | 19 | | 2470 | |  | | Thudi et al. (2011) | |
| CaM0955 | | EI868108 | | CaLG02; 62.75 | | CAH1034D19 | | ctg14 | | 40 | | 5200 | | AB, FW | |  | |
| CaM0958 | | EI868152 | | CaLG07; 18.28 | | CAH1034N03 | | ctg2064 | | 35 | | 4550 | |  | | Thudi et al. (2011) | |
| CaM0964 | | EI868215 | | CaLG07; 18.28 | | CAH1038H23 | | ctg99 | | 88 | | 11440 | |  | | Thudi et al. (2011) | |
| CaM0998 | | EI868800 | | CaLG07; 18.28 | | CAH1032E07 | | ctg1955 | | 17 | | 2210 | | Plant height (9.46) | | Varshney et al. 2014 | |
| CaM1007 | | EI869042 | | CaLG03; 5.96 | | CAH1033A07 | | ctg27 | | 288 | | 37440 | |  | | Thudi et al. (2011) | |
| CaM1016 | | EI869152 | | CaLG05; 97.33 | | CAH1033G09 | | ctg74 | | 157 | | 20410 | |  | | Thudi et al. (2011) | |
| CaM1020 | | EI869214 | | CaLG03; 30.30 | | CAH1038C07 | | ctg43 | | 119 | | 15470 | | Beta carotene QTL2 (on LG3B) | | Abbo et al. (2005) | |
| CaM1024 | | EI869285 | | CaLG03; 30.30 | | CAH1038O13 | | ctg42 | | 263 | | 34190 | |  | | Thudi et al. (2011) | |
| CaM1036 | | EI869671 | | CaLG05; 81.38 | | CAH1038G08 | | ctg30 | | 133 | | 17290 | |  | | Thudi et al. (2011) | |
| CaM1042 | | EI869844 | | CaLG03; 61.93 | | CAH1032J01 | | ctg46 | | 126 | | 16380 | |  | | Thudi et al. (2011) | |
| CaM1068 | | EI870413 | | CaLG05; 105.64 | | CAH1035J08 | | ctg1229 | | 112 | | 14560 | |  | | Thudi et al. (2011) | |
| CaM1072 | | EI870534 | | CaLG05; 107.40 | | CAH1033B06 | | ctg0 | | - | | - | |  | | Thudi et al. (2011) | |
| CaM1079 | | EI870699 | | CaLG03; 10.94 | | CAH1033B04 | | ctg870 | | 5 | | 650 | |  | | Thudi et al. (2011) | |
| CaM1084 | | EI870809 | | CaLG06; 78.49 | | CAH1037G07 | | ctg1776 | | 260 | | 33800 | |  | | Thudi et al. (2011) | |
| CaM1089 | | EI870908 | | CaLG06; 78.49 | | CAH1037H17 | | ctg137 | | 34 | | 4420 | |  | | Thudi et al. (2011) | |
| CaM1098 | | EI871052 | | CaLG05; 100.02 | | CAH1036A22 | | ctg334 | | 38 | | 4940 | |  | | Thudi et al. (2011) | |
| CaM1101 | | EI871128 | | CaLG06; 92.62 | | CAH1036O22 | | ctg0 | | - | | - | |  | | Thudi et al. (2011) | |
| CaM1117 | | EI871452 | | CaLG06; 92.62 | | CAH1046D05 | | ctg1047 | | 70 | | 9100 | |  | | Thudi et al. (2011) | |
| **Marker** | | **Genbank ID** | | **Position on genetic map (Linkage group; distance in cM)** | | **BAC clone ID** | | **Contig ID** | | **No of clones/ contig** | | **Size (kb)** | | **QTL information (Trait; Phenotypic variation %)** | | **Reference** | |
| CaM1122 | | EI871582 | | CaLG03; 40.57 | | CAH1049N21 | | ctg0 | | - | | - | | Beta carotene QTL2 (on LG3B) | | Abbo et al. (2005) | |
| CaM1125 | | EI871652 | | CaLG06; 76.16 | | CAH1040K07 | | ctg569 | | 63 | | 8190 | | Days to 50% flowering (6.7723) | | Varshney et al. 2014 | |
| CaM1129 | | EI871710 | | CaLG03; 27.19 | | CAH1040E21 | | ctg1229 | | 112 | | 14560 | | Beta carotene QTL2 (on LG3B) | | Abbo et al. (2005) | |
| CaM1132 | | EI871804 | | CaLG03; 19.93 | | CAH1041G15 | | ctg373 | | 20 | | 2600 | | Beta carotene QTL2 (on LG3B) | | Abbo et al. (2005) | |
| CaM1135 | | EI871868 | | CaLG02; 32.05 | | CAH1041C17 | | ctg1390 | | 2 | | 260 | | AB, FW | |  | |
| CaM1149 | | EI872207 | | CaLG03; 0.92 | | CAH1040O16 | | ctg703 | | 6 | | 780 | |  | | Thudi et al. (2011) | |
| CaM1158 | | EI872397 | | CaLG04; 60.76 | | CAH1040A22 | | ctg327 | | 38 | | 4940 | |  | | Thudi et al. (2011) | |
| CaM1159 | | EI872401 | | CaLG07; 52.89 | | CAH1040C08 | | ctg1893 | | 342 | | 44460 | |  | | Thudi et al. (2011) | |
| CaM1193 | | EI873181 | | CaLG07; 52.89 | | CAH1039N19 | | ctg0 | | - | | - | |  | | Thudi et al. (2011) | |
| CaM1214 | | EI873621 | | CaLG07; 52.89 | | CAH1041B24 | | ctg7 | | 500 | | 65000 | |  | | Thudi et al. (2011) | |
| CaM1218 | | EI873716 | | CaLG05; 82.76 | | CAH1042E03 | | ctg0 | | - | | - | |  | | Thudi et al. (2011) | |
| CaM1228 | | EI873918 | | CaLG05; 82.14 | | CAH1042G19 | | ctg1798 | | 138 | | 17940 | |  | | Thudi et al. (2011) | |
| CaM1232 | | EI874002 | | CaLG05; 82.14 | | CAH1043G17 | | ctg1714 | | 36 | | 4680 | |  | | Thudi et al. (2011) | |
| CaM1238 | | EI874129 | | CaLG05; 107.95 | | CAH1043O14 | | ctg500 | | 249 | | 32370 | |  | | Thudi et al. (2011) | |
| CaM1239 | | EI874140 | | CaLG06; 42.44 | | CAH1043A12 | | ctg0 | | - | | - | |  | | Thudi et al. (2011) | |
| CaM1257 | | EI874432 | | CaLG06; 42.44 | | CAH1043F23 | | ctg397 | | 133 | | 17290 | |  | | Thudi et al. (2011) | |
| CaM1258 | | EI874464 | | CaLG06; 24.37 | | CAH1043L21 | | ctg325 | | 19 | | 2470 | |  | | Varshney et al. 2014 | |
| **Marker** | | **Genbank ID** | | **Position on genetic map (Linkage group; distance in cM)** | | **BAC clone ID** | | **Contig ID** | | **No of clones/ contig** | | **Size (kb)** | | **QTL information (Trait; Phenotypic variation %)** | | **Reference** | |
| CaM1274 | | EI874745 | | CaLG08; 53.85 | | CAH1043L21 | | ctg325 | | 19 | | 2470 | |  | | Varshney et al. 2014 | |
| CaM1328 | | EI875795 | | CaLG04; 65.36 | | CAH1048K20 | | ctg1769 | | 230 | | 29900 | |  | | Varshney et al. 2014 | |
| CaM1337 | | EI875998 | | CaLG04; 65.36 | | CAH1048J15 | | ctg572 | | 9 | | 1170 | |  | | Varshney et al. 2014 | |
| CaM1354 | | EI876285 | | CaLG04; 65.36 | | CAH1047J10 | | ctg330 | | 31 | | 4030 | | Beta carotene QTL2 (on LG3B) | | Abbo et al. (2005) | |
| CaM1358 | | EI876349 | | CaLG04; 65.36 | | CAH1046H04 | | ctg822 | | 7 | | 910 | | Beta carotene QTL2 (on LG3B) | | Abbo et al. 2006 | |
| CaM1360 | | EI876390 | | CaLG04; 65.36 | | CAH1046P08 | | ctg1893 | | 342 | | 44460 | |  | | Varshney et al. 2014 | |
| CaM1372 | | EI876550 | | CaLG05; 34.71 | | CAH1044C05 | | ctg16 | | 293 | | 38090 | |  | | Varshney et al. 2014 | |
| CaM1376 | | EI876669 | | CaLG03; 42.21 | | CAH1044K23 | | ctg1968 | | 47 | | 6110 | | Plant height (9.46) | | Varshney et al. 2014 | |
| CaM1377 | | EI876701 | | CaLG03; 42.21 | | CAH1049C07 | | ctg397 | | 133 | | 17290 | |  | | Varshney et al. 2014 | |
| CaM1389 | | EI877137 | | CaLG05; 33.48 | | CAH1044L15 | | ctg222 | | 75 | | 9750 | |  | | Varshney et al. 2014 | |
| CaM1399 | | EI877361 | | CaLG08; 53.70 | | CAH1044P02 | | ctg1994 | | 133 | | 17290 | |  | | Varshney et al. 2014 | |
| CaM1402 | | EI877412 | | CaLG06; 22.03 | | CAH1049J10 | | ctg0 | | - | | - | |  | | Varshney et al. 2014 | |
| CaM1417 | | EI877674 | | CaLG07; 76.68 | | CAH1048N18 | | ctg386 | | 24 | | 3120 | | Seeds/pod (6.23), DSI (5.74-6.23) | | Varshney et al. 2014 | |
| **Marker** | | **Genbank ID** | | **Position on genetic map (Linkage group; distance in cM)** | | **BAC clone ID** | | **Contig ID** | | **No of clones/contig** | | **Size (kb)** | | **QTL information (Trait; Phenotypic variation %)** | | **Reference** | |
| CaM1431 | | EI877994 | | CaLG06; 31 | | CAH1053K03 | | ctg702 | | 8 | | 1040 | |  | | Varshney et al. 2014 | |
| CaM1451 | | EI878481 | | CaLG06; 31 | | CAH1053B15 | | ctg163 | | 22 | | 2860 | |  | | Varshney et al. 2014 | |
| CaM1469 | | EI878861 | | CaLG07; 60.62 | | CAH1055I02 | | ctg1714 | | 36 | | 4680 | | Plant height (5.12) | | Varshney et al. 2014 | |
| CaM1477 | | EI878939 | | CaLG07; 60.62 | | CAH1053H08 | | ctg702 | | 8 | | 1040 | |  | | Varshney et al. 2014 | |
| CaM1496 | | EI879269 | | CaLG07; 60.62 | | CAH1061E06 | | ctg308 | | 18 | | 2340 | |  | | Varshney et al. 2014 | |
| CaM1497 | | EI879270 | | CaLG07; 60.62 | | CAH1061E08 | | ctg308 | | 18 | | 2340 | |  | | Varshney et al. 2014 | |
| CaM1502 | | EI879364 | | CaLG07; 60.62 | | CAH1061G08 | | ctg4 | | 74 | | 9620 | |  | | Varshney et al. 2014 | |
| CaM1506 | | EI879466 | | CaLG07; 60.62 | | CAH1061J19 | | ctg370 | | 47 | | 6110 | |  | | Varshney et al. 2014 | |
| CaM1515 | | EI879617 | | CaLG07; 60.62 | | CAH1061J19 | | ctg370 | | 47 | | 6110 | |  | |  | |
| CaM1517 | | EI879655 | | CaLG05; 26.39 | | CAH1061J19 | | ctg370 | | 47 | | 6110 | |  | | Varshney et al. 2014 | |
| CaM1529 | | EI879902 | | CaLG05; 26.39 | | CAH1066K23 | | ctg869 | | 4 | | 520 | |  | | Varshney et al. 2014 | |
| CaM1530 | | EI879913 | | CaLG06; 31.83 | | CAH1066M23 | | ctg554 | | 12 | | 1560 | |  | | Varshney et al. 2014 | |
| CaM1536 | | EI880145 | | CaLG06; 31.83 | | CAH1066I02 | | ctg132 | | 43 | | 5590 | |  | | Varshney et al. 2014 | |
| CaM1542 | | EI880315 | | CaLG06; 31.83 | | CAH1065G09 | | ctg403 | | 8 | | 1040 | |  | | Varshney et al. 2014 | |
| CaM1545 | | EI880390 | | CaLG06; 31.83 | | CAH1066F13 | | ctg269 | | 52 | | 6760 | |  | | Varshney et al. 2014 | |
| CaM1551 | | EI880500 | | CaLG06; 31.83 | | CAH1066J09 | | ctg1875 | | 45 | | 5850 | |  | | Varshney et al. | |
| **Marker** | | **Genbank ID** | | **Position on genetic map (Linkage group; distance in cM)** | | **BAC clone ID** | | **Contig ID** | | **No of clones/ contig** | | **Size (kb)** | | **QTL information (Trait; Phenotypic variation %)** | | **Reference** | |
|  | |  | |  | |  | |  | |  | |  | |  | |  | |
| CaM1567 | | EI880807 | | CaLG07; 49.82 | | CAH1067C05 | | ctg374 | | 195 | | 25350 | |  | | Varshney et al. 2014 | |
| CaM1568 | | EI880807 | | CaLG03; 39.56 | | CAH1067C05 | | ctg374 | | 195 | | 25350 | | Yield (7.01), Biomass (5.62) | | Varshney et al. 2014 | |
| CaM1581 | | EI881112 | | CaLG03; 39.56 | | CAH1064J19 | | ctg392 | | 33 | | 4290 | |  | | Varshney et al. 2014 | |
| CaM1590 | | EI881234 | | CaLG03; 39.56 | | CAH1065P19 | | ctg417 | | 17 | | 2210 | |  | | Varshney et al. 2014 | |
| CaM1591 | | EI881258 | | CaLG03; 39.56 | | CAH1065F05 | | ctg10 | | 237 | | 30810 | |  | | Varshney et al. 2014 | |
| CaM1607 | | EI881648 | | CaLG03; 39.56 | | CAH1065N02 | | ctg462 | | 96 | | 12480 | |  | | Varshney et al. 2014 | |
| CaM1620 | | EI882086 | | CaLG03; 39.56 | | CAH1065N02 | | ctg462 | | 96 | | 12480 | |  | | Varshney et al. 2014 | |
| CaM1637 | | EI882442 | | CaLG03; 39.56 | | CAH1064B05 | | ctg328 | | 20 | | 2600 | |  | | Varshney et al. 2014 | |
| CaM1648 | | EI882653 | | CaLG03; 39.56 | | CAH1067I06 | | ctg1748 | | 89 | | 11570 | |  | | Varshney et al. 2014 | |
| CaM1658 | | EI882831 | | CaLG03; 39.56 | | CAH1064J08 | | ctg101 | | 26 | | 3380 | |  | | Varshney et al. 2014 | |
| CaM1666 | | EI882956 | | CaLG03; 39.56 | | CAH1063B15 | | ctg2019 | | 32 | | 4160 | |  | | Varshney et al. 2014 | |
| CaM1668 | | EI883028 | | CaLG03; 39.56 | | CAH1063B15 | | ctg2019 | | 32 | | 4160 | |  | | Varshney et al. 2014 | |
| CaM1684 | | EI883208 | | CaLG04; 75.64 | | CAH1062C02 | | ctg56 | | 146 | | 18980 | | 100-seed weight (4.17-6.39); DSI (5.63), Harvest index (7.25) | | Varshney et al. 2014 | |
| CaM1687 | | EI883262 | | CaLG07; 66.61 | | CAH1062M06 | | ctg188 | | 48 | | 6240 | |  | | Varshney et al. | |
| **Marker** | | **Genbank ID** | | **Position on genetic map (Linkage group; distance in cM)** | | **BAC clone ID** | | **Contig ID** | | **No of clones/ contig** | | **Size (kb)** | | **QTL information (Trait; Phenotypic variation %)** | | **Reference** | |
| CaM1702 | | EI883530 | | CaLG01; 70.44 | | CAH1062D11 | | ctg98 | | 242 | | 31460 | |  | | Varshney et al. 2014 | |
| CaM1714 | | EI883772 | | CaLG01; 70.44 | | CAH1068C15 | | ctg572 | | 9 | | 1170 | |  | | Varshney et al. 2014 | |
| CaM1722 | | EI883829 | | CaLG01; 70.44 | | CAH1068O21 | | ctg172 | | 33 | | 4290 | |  | | Varshney et al. 2014 | |
| CaM1742 | | EI884153 | | CaLG01; 71.25 | | CAH1068E20 | | ctg94 | | 60 | | 7800 | |  | | Varshney et al. 2014 | |
| CaM1750 | | EI884351 | | CaLG01; 71.25 | | CAH1069A18 | | ctg76 | | 116 | | 15080 | |  | |  | |
| CaM1753 | | EI884410 | | CaLG03; 28.59 | | CAH1069M12 | | ctg1893 | | 342 | | 44460 | |  | | Varshney et al. 2014 | |
| CaM1760 | | EI884491 | | CaLG06; 77.35 | | CAH1051M10 | | ctg1485 | | 1674 | | 217620 | | Shoot dry weight (5.81); Plant height 5.94-12.16); Primary branches (8.48), | | Varshney et al. 2014 | |
| CaM1763 | | EI884523 | | CaLG06; 77.35 | | CAH1068F09 | | ctg281 | | 34 | | 4420 | |  | | Varshney et al. 2014 | |
| CaM1765 | | EI884535 | | CaLG03; 30.59 | | CAH1068H13 | | ctg45 | | 348 | | 45240 | | Root dry weight (9.49%) | | Varshney et al. 2014 | |
| CaM1767 | | EI884583 | | CaLG03; 36.80 | | CAH1050D03 | | ctg0 | | - | | - | |  | | Varshney et al. 2014 | |
| CaM1782 | | EI884797 | | CaLG03; 36.80 | | CAH1069N05 | | ctg99 | | 88 | | 11440 | |  | | Varshney et al. 2014 | |
| CaM1790 | | EI884897 | | CaLG06; 28.49 | | CAH1069N23 | | ctg0 | | - | | - | |  | | Varshney et al. 2014 | |
| CaM1806 | | EI885134 | | CaLG05; 24.94 | | CAH1069J06 | | ctg452 | | 22 | | 2860 | |  | | Varshney et al. 2014 | |
| CaM1809 | | EI885181 | | CaLG05; 24.94 | | CAH1068D04 | | ctg74 | | 157 | | 20410 | |  | | Varshney et al. 2014 | |
| **Marker** | | **Genbank ID** | | **Position on genetic map (Linkage group; distance in cM)** | | **BAC clone ID** | | **Contig ID** | | **No of clones/ contig** | | **Size (kb)** | | **QTL information (Trait; Phenotypic variation %)** | | **Reference** | |
| CaM1826 | | EI885456 | | CaLG06; 30.53 | | CAH1071M23 | | ctg19 | | 130 | | 16900 | | Root dry weight (5.47%) | | Varshney et al. 2014 | |
| CaM1827 | | EI885508 | | CaLG06; 30.53 | | CAH1071I21 | | ctg169 | | 287 | | 37310 | |  | | Varshney et al. 2014 | |
| CaM1835 | | EI885651 | | CaLG03; 31.33 | | CAH1070E13 | | ctg168 | | 149 | | 19370 | |  | | Varshney et al. 2014 | |
| CaM1843 | | EI885751 | | CaLG03; 31.63 | | CAH1054G09 | | ctg232 | | 79 | | 10270 | | Root dry weight (9.49%) | | Varshney et al. 2014 | |
| CaM1851 | | EI885874 | | CaLG03; 29.74 | | CAH1070M18 | | ctg388 | | 15 | | 1950 | |  | | Varshney et al. 2014 | |
| CaM1853 | | EI885911 | | CaLG03; 29.74 | | CAH1070E02 | | ctg353 | | 26 | | 3380 | |  | | Varshney et al. 2014 | |
| CaM1855 | | EI885969 | | CaLG06; 7.50 | | CAH1070O10 | | ctg151 | | 60 | | 7800 | |  | | Varshney et al. 2014 | |
| CaM1868 | | EI886327 | | CaLG06; 7.50 | | CAH1071F03 | | ctg355 | | 30 | | 3900 | |  | | Varshney et al. 2014 | |
| CaM1903 | | EI887062 | | CaLG06; 7.50 | | CAH1071P02 | | ctg485 | | 20 | | 2600 | | RT ratio (6.98%); | | Varshney et al. 2014 | |
| CaM1905 | | EI887129 | | CaLG03; 44.97 | | CAH1069L12 | | ctg1893 | | 342 | | 44460 | |  | | Varshney et al. 2014 | |
| CaM1907 | | EI887228 | | CaLG02; 39.59 | | CAH1054P08 | | ctg0 | | - | | - | |  | | Varshney et al. 2014 | |
| CaM1908 | | EI887228 | | CaLG02; 34.07 | | CAH1054P08 | | ctg0 | | - | | - | |  | | Varshney et al. 2014 | |
| CaM1918 | | EI887393 | | CaLG08; 40.85 | | CAH1052A13 | | ctg0 | | - | | - | | Days to 50% flowering (9.1-10.5); Days to maturity (8.76-12.14) | | Varshney et al. 2014 | |
| CaM1942 | | EI887759 | | CaLG07; 59.69 | | CAH1072I14 | | ctg190 | | 492 | | 63960 | |  | | Varshney et al. | |
| **Marker** | | **Genbank ID** | | **Position on genetic map (Linkage group; distance in cM)** | | **BAC clone ID** | | **Contig ID** | | **No of clones/contig** | | **Size (kb)** | | **QTL information (Trait; Phenotypic variation %)** | | **Reference** | |
| CaM1943 | | EI887765 | | CaLG06; 19.52 | | CAH1072K02 | | ctg517 | | 11 | | 1430 | |  | | Varshney et al. 2014 | |
| CaM1973 | | EI888255 | | CaLG03; 34.99 | | CAH1054D13 | | ctg300 | | 27 | | 3510 | |  | | Varshney et al. 2014 | |
| CaM1975 | | EI888263 | | CaLG03; 34.99 | | CAH1054F11 | | ctg520 | | 27 | | 3510 | |  | | Varshney et al. 2014 | |
| CaM1977 | | EI888347 | | CaLG05; 42.74 | | CAH1072F09 | | ctg651 | | 27 | | 3510 | |  | | Varshney et al. 2014 | |
| CaM2026 | | EI889218 | | CaLG05; 16.65 | | CAH1059E21 | | ctg1779 | | 110 | | 14300 | |  | | Varshney et al. 2014 | |
| CaM2029 | | EI889255 | | CaLG05; 42.98 | | CAH1059M17 | | ctg2 | | 62 | | 8060 | |  | | Varshney et al. 2014 | |
| CaM2031 | | EI889290 | | CaLG03; 24.71 | | CAH1060E03 | | ctg1952 | | 8 | | 1040 | |  | | Varshney et al. 2014 | |
| CaM2032 | | EI889344 | | CaLG03; 24.71 | | CAH1060M23 | | ctg462 | | 96 | | 12480 | |  | | Varshney et al. 2014 | |
| CaM2036 | | EI889403 | | CaLG03; 24.71 | | CAH1060J11 | | ctg912 | | 7 | | 910 | |  | | Varshney et al. 2014 | |
| CaM2041 | | EI889572 | | CaLG07; 77.18 | | CAH1059I16 | | ctg44 | | 217 | | 28210 | |  | | Varshney et al. 2014 | |
| CaM2045 | | EI889642 | | CaLG07; 77.18 | | CAH1060E20 | | ctg0 | | - | | - | |  | | Varshney et al. 2014 | |
| CaM2049 | | EI889687 | | CaLG07; 77.18 | | CAH1060M14 | | ctg607 | | 9 | | 1170 | |  | | Varshney et al. 2014 | |
| CaM2063 | | EI889995 | | CaLG08; 64.33 | | CAH1060H09 | | ctg0 | | - | | - | |  | | Varshney et al. 2014 | |
| CaM2064 | | EI890022 | | CaLG08; 64.33 | | CAH1060L17 | | ctg607 | | 9 | | 1170 | |  | | Varshney et al. 2014 | |
| CaM2074 | | EI890217 | | CaLG04; 72.89 | | CAH1059D24 | | ctg1751 | | 123 | | 15990 | |  | | Varshney et al. 2014 | |
| **Marker** | | **Genbank ID** | | **Position on genetic map (Linkage group; distance in cM)** | | **BAC clone ID** | | **Contig ID** | | **No of clones/contig** | | **Size (kb)** | | **QTL information (Trait; Phenotypic variation %)** | | **Reference** | |
| CaM2085 | | EI890549 | | CaLG04; 72.89 | | CAH1058E11 | | ctg64 | | 547 | | 71110 | |  | | Varshney et al. 2014 | |
| CaM2093 | | EI890698 | | CaLG04; 69.27 | | CAH1058F19 | | ctg365 | | 99 | | 12870 | |  | | Varshney et al. 2014 | |
| CaM2094 | | EI890711 | | CaLG07; 60.25 | | CAH1058H21 | | ctg439 | | 35 | | 4550 | |  | | Varshney et al. 2014 | |
| CaM2095 | | EI890753 | | CaLG03; 40.30 | | CAH1058A06 | | ctg810 | | 9 | | 1170 | |  | | Varshney et al. 2014 | |
| CaM2098 | | EI890787 | | CaLG05; 15.86 | | CAH1058G18 | | ctg22 | | 60 | | 7800 | |  | | Varshney et al. 2014 | |
| CaM2102 | | EI890831 | | CaLG03; 40.90 | | CAH1057B06 | | ctg269 | | 52 | | 6760 | |  | | Varshney et al. 2014 | |
| CaM2131 | | EI891567 | | CaLG03; 40.90 | | CAH1059L21 | | ctg238 | | 79 | | 10270 | |  | | Varshney et al. 2014 | |
| CaM2155 | | EI892153 | | CaLG07; 59.12 | | CAH1063N17 | | ctg271 | | 86 | | 11180 | |  | | Varshney et al. 2014 | |
| CaM2158 | | EI892187 | | CaLG05; 25.59 | | CAH1063C24 | | ctg1552 | | 69 | | 8970 | |  | | Varshney et al. 2014 | |
| CaM2162 | | EI892252 | | CaLG05; 25.59 | | CAH1063O14 | | ctg246 | | 14 | | 1820 | |  | | Varshney et al. 2014 | |
| CaM2168 | | EI892368 | | CaLG05; 25.59 | | CAH1050H16 | | ctg1776 | | 260 | | 33800 | |  | | Varshney et al. 2014 | |
| CaM2174 | | EI892426 | | CaLG03; 43.04 | | CAH1069D07 | | ctg0 | | - | | - | |  | | Varshney et al. 2014 | |
| CaM2181 | | EI892588 | | CaLG03; 31.21 | | CAH1072A09 | | ctg116 | | 71 | | 9230 | |  | | Varshney et al. 2014 | |
| **Marker** | | **Genbank ID** | | **Position on genetic map (Linkage group; distance in cM)** | | **BAC clone ID** | | **Contig ID** | | **No of clones/contig** | | **Size (kb)** | | **QTL information (Trait; Phenotypic variation %)** | | **Reference** | |
| CaM2186 | | EI892642 | | CaLG03; 31.21 | | CAH1072K09 | | ctg510 | | 24 | | 3120 | |  | | Varshney et al. 2014 | |
| CaM2187 | | EI892699 | | CaLG08; 27.87 | | CAH1072H06 | | ctg29 | | 245 | | 31850 | | Harvest index (5.1 6.4789); Days to 50% flowering (8.5-26.8); Biomass (8.2 - 10.9531); Days to maturity (8.76-18.14); Harvest index (9.0467); Plant height (14.72) | | Varshney et al. 2014 | |
| CaM2189 | | EI892739 | | CaLG08; 71.50 | | CAH1072N24 | | ctg169 | | 287 | | 37310 | |  | | Varshney et al. 2014 | |
