# Supplementary material for: Integrated physical, genetic and genome map of chickpea (Cicer arietinum L.)
Source: Funct Integr Genomics. 2014 Mar 8;14(1):59–73. doi: 10.1007/s10142-014-0363-6 (PMC4273598; doi:10.1007/s10142-014-0363-6)
Supplement: Supplementary file 18 — The distribution of repetitive sequences in 5,3316 BES used for their mapping on chickpea genome sequence (DOCX 17 kb) [file 10142_2014_363_MOESM10_ESM.docx]

**Supplementary Table 2: The distribution of repetitive sequences in 53,316 BES used for their mapping on chickpea genome sequence**

| **Repeat element** | **Number of elements** | **Length occupied (bp)** | **Sequence (%)** |
| --- | --- | --- | --- |
| SINEs | 16 | 604 | 0 |
| LINEs | 1,135 | 2,28,118 | 0.6 |
| LTR elements | 5,0481 | 2,24,67,920 | 59.44 |
| DNA elements | 6,588 | 18,59,666 | 4.92 |
| Unclassified | 7,501 | 12,48,664 | 3.3 |
| Total interspersed repeats | 2,480 | 2,58,04,972 | 68.27 |
| Satellites | 537 | 2,46,286 | 0.65 |
| Simple repeats | 2,790 | 5,03,995 | 1.33 |
| Low complexity | 2,921 | 1,34,640 | 0.36 |
